# Warming outweighs nitrogen deposition in shaping rhizosphere microbial structure involved in carbon, nitrogen, and phosphorus cycling in *Ambrosia trifida*

**DOI:** 10.3389/fpls.2026.1686326

**Published:** 2026-02-10

**Authors:** Ke Xu, Ping Guan, Wanyu Du, Huiyu Zeng, Meishan Chen, Zhenhuan Lv, Yanhan Liu, Meini Shao, Bo Qu

**Affiliations:** 1 Liaoning Key Laboratory of Biological Invasions and Global Changes, College of Biological Science and Biotechnology, Shenyang Agricultural University, Shenyang, China; 2 College of Science, Shenyang Agricultural University, Shenyang, China; 3 Chaoyang Ecology and Environment Service Center, Chaoyang, China

**Keywords:** *Ambrosia trifida*, global warming, nitrogen deposition, root exudates, soil microorganism

## Abstract

**Introduction:**

*Ambrosia trifida*, a harmful invasive plant, poses significant ecological and economic threats and is expected to spread further under future warming and nitrogen deposition scenarios. According to plant-soil feedback and enhanced mutualist hypothesis, invasive plants may gain a competitive edge by recruiting specific microorganisms. However, little is known about the composition and functional potential of its rhizosphere microbiome.

**Methods:**

In this study, we combined metagenomics with widely targeted metabolomics to investigate the interactions between root exudates and soil microbial communities under experimental warming and nitrogen deposition.

**Results and discussion:**

The results showed that warming and nitrogen addition together promoted biomass accumulation. And their combination enhanced soil nutrient content. Warming increased the abundance of functional genes involved in carbon fixation (e.g., *acs, acsA, PCCA, MUT*), whereas nitrogen addition suppressed nitrification and denitrification genes. Warming also enhanced the abundance of genes related to inorganic phosphate solubilization (*ppk, ppx*), phosphorus mineralization (*phnPP, phnF, glpQ*), and phosphorus transport (*pstBC, ugpABCE*). Functionally, warming increased the relative abundance of beneficial taxa such as *Sphingomicrobium, Massilia*, and *Nocardioides*, while reducing *Pseudomonas, Trinickia*, and *Rhizomicrobium*. Nitrogen deposition had a comparatively weaker effect on the functional microbial community. Correlation analysis between metabolites and functional genes suggested that alkaloids, organic acids, and phenolic compounds may be key drivers of microbial functional shifts. Overall, our findings demonstrate that warming has a greater influence than nitrogen deposition on shaping the rhizosphere soil microbial community and enhancing nutrient cycling functions, potentially increasing the risk of *A. trifida* invasion under future climate change.

## 1 Introduction

The invasion of alien plant species is changing the structure and composition of biological communities at an unprecedented rate, thereby altering the global environment (Guido and Pillar, 2017; Sardans et al., 2017). Due to the characteristics of rapid growth, strong resprouting ability, strong resource competition ability, fast reproduction speed and high resource consumption of alien species (Davis et al., 2000),alien species threaten the survival of native species and reduces the biodiversity and functions of ecosystems (Divíšek et al., 2018).

High competitiveness seems to be the key to successful plant invasion, reflecting the powerful ability of invasive plants to acquire resources that are often limited in growth (Seabloom et al., 2003; Lidbury et al., 2016). The enhanced mutualisms hypothesis suggests that alien plants often establish beneficial symbiotic relationships with soil microbial communities in the invaded range, and these mutualisms can play an important role in facilitating invasion (Reinhart and Callaway, 2006). For example, *Mikania micrantha* can increase the availability of inorganic nitrogen by increasing the abundance of ammonia oxidizing archaea and bacteria (Liu et al., 2020) and increase the available phosphorus content in soil by recruiting phosphorus-solubilizing *Pseudomonas* (Yin et al., 2020). Plant invasion profoundly affects soil nutrient cycling and microbial communities in ecosystems (Liao et al., 2008; Li et al., 2022), therefore, it is necessary to understand how soil microorganisms alter carbon, nitrogen, and phosphorus cycling during plant invasion.

Rising temperatures, resulting from climate change, may facilitate plant invasion by enhancing the proliferation and dispersal of invasive species (Walther et al., 2009; Lu et al., 2013). Increased nitrogen deposition exacerbates the introduction and spreads of invasive plant species, giving them a competitive advantage (Dukes and Mooney, 1999; Clark and Tilman, 2008) and reducing plant diversity (Ehrenfeld, 2003; Field et al., 2014). Climate change can alter soil microbial assemblages in ways that reshape plant–microbiome interactions, potentially affecting the performance and invasion success of non-native plant species (Crowther et al., 2019). Yet, the responses of soil microbiota to climatic shifts often diverge markedly from those of their host plants, introducing significant uncertainty into predictions of microbial community dynamics under future climate scenarios (Zhou et al., 2020). Therefore, more research is needed to fully understand the complex interactions between invasive plants and soil microbial communities, and to develop effective management strategies (Coats and Rumpho, 2014);. The new weapon hypothesis (NWH) theory suggests that the success of plant invasion depends on the ability to release new plant chemicals, including allelopathic and antibacterial root exudates, into the invaded community (Callaway and Ridenour, 2004). Under global climate change, rising temperatures can increase the length of plant growth seasons and plant biomass production, thereby enhancing the inputs of root exudates and aboveground and underground litter into the soil (Dusenge et al., 2018). Nitrogen deposition directly alleviates nutrient limitations in plants, leading to a reduction in carbon input from root exudates and a decrease in soil microbial quantity and activity (Xiong et al., 2020). The interaction between global warming and nitrogen deposition is complex, potentially leading to synergistic or antagonistic effects. However, studies investigating how these two factors jointly affect soil nutrients—and the mechanisms by which they influence rhizosphere microbial communities of invasive plants—remain limited.

*Ambrosia trifida*, an annual weed native to North America and belonging to the Asteraceae family, has become a notorious invasive species worldwide (Qin et al., 2014). Over the past 200 years, *A. trifida* has spread from Eurasia and Australia to 40 countries (Xian et al., 2023). In China, the existence of *A. trifida* was first recorded in Tieling in the 1930s, and subsequently expanded to 17 provinces, from Heilongjiang in the northeast to Sichuan in the southwest and Xinjiang in the northwest (Wang et al., 2022). The invasion of *A. trifida* has led to a decrease in the stability and species diversity of natural ecosystems, while also causing serious damage to farmland (Li et al., 2022). One of the main reasons why *A. trifida* is so invasive is its ability to quickly adapt to new environments, especially to new soil habitats. Therefore, it is necessary to develop effective strategies and theoretical frameworks *A. trifida* (Schaffner et al., 2020). Our previous research found significant differences in the composition of organic acids, phenolic acids, and lipids in the root exudates of *A. trifida* under nitrogen and temperature increasing conditions (Xu et al., 2023). Base on these, the successful invasion of *A. trifida* may be facilitated by its ability to recruit beneficial microorganisms through root exudates, thereby enhancing soil nutrient cycling and promoting establishment in new environments.

To investigate whether *A. trifida* recruits beneficial rhizosphere microorganisms through root exudates and gains a competitive advantage under climate change, we combined metagenomics and widely targeted metabolomics in experiments simulating global warming and nitrogen deposition. This study sheds light on the interaction mechanisms between *A. trifida* and soil microorganisms, revealing the microbial basis of its successful invasion and providing a theoretical foundation for the prevention and control of invasive species.

## 2 Materials and methods

### 2.1 Plant cultivation

The experimental site is located at the teaching and research base of Shenyang Agricultural University in Shenyang, Liaoning Province (41°49′N, 123°34′E). The experiment includes one control group (C) and three treatment groups: warming (W), nitrogen addition (N), and combined warming and nitrogen addition (WN), with 30 replicates per group. Wild plants with uniform growth were selected and transplanted into pots by the end of April.

Warming was simulated using infrared radiation heaters, which maintained a temperature increase of approximately 2°C compared to its surroundings. Nitrogen deposition was simulated via wet deposition, with a total application rate of 5 g N m^−2^ yr^−1^. The added nitrogen was a composite of ammonium, nitrate, and amide nitrogen in a 1:1:1 ratio, applied four times at one-week intervals (Ren et al., 2021).

### 2.2 Plant trait measurement

Measurements were taken separately during the seedling (S) and mature (M) stages of the plant. To get dry weight, thirty replicate roots, stems, and leaves were dehydrated in an oven at 70°C for 24 h. Add the weight of root, stem, and leave together to determine the total dry weight of the plant. Next, calculate the root mass fraction (root dry mass/total plant dry mass, RMF), stem mass fraction (stem dry mass/total plant dry mass, SMF), and leaf mass fraction (leaf dry mass/total plant dry mass, LMF) separately.

### 2.3 Soil collection

The entire plant root was carefully excavated, and loosely attached bulk soil was gently shaken off. Approximately 15 g of root-adhering soil (rhizosphere soil) was collected using sterile scissors and transferred into a 25 mL centrifuge tube containing sterile 0.86% NaCl solution. Four replicate soil samples were collected for each treatment.

The samples were incubated on ice for 30 minutes and gently shaken every 5 minutes. After incubation, plant roots were removed, and the suspension was centrifuged at 4000 g for 30 minutes at 4°C. The supernatant was discarded, and the resulting soil pellet was transferred to a sterile Eppendorf (EP) tube and stored at –20°C for further analysis.

### 2.4 Measurement of soil physicochemical properties

Soil pH was measured using a pH meter (Mettler Toledo Instruments, Shanghai, China) in a 1:2 (w/v) soil-to-water suspension.

Soil organic carbon (SOC) content was determined from 0.25 g of air-dried soil using the potassium dichromate–sulfuric acid oxidation method.

Total nitrogen (TN) was measured using the Kjeldahl method.

Total phosphorus (TP) was determined via NaOH-alkaline fusion followed by molybdenum-antimony colorimetric spectrophotometry.

Total potassium (TK) was measured using flame photometry after NaOH fusion.

### 2.5 Metagenome DNA extraction and shotgun sequencing

Total microbial genomic DNA were extracted using the OMEGA Soil DNA Kit (D5625-01). The quantity and quality of extracted DNAs were measured using a NanoDrop ND-1000 spectrophotometer (Thermo Fisher Scientific, Waltham, MA,USA) and agarose gel electrophoresis, respectively. The extracted microbial DNA was processed to construct metagenome shotgun sequencing libraries with insert sizes of 400 bp by using Illumina TruSeq Nano DNA LT Library Preparation Kit. Each library was sequenced by Illumina HiSeq X-ten platform (Illumina, USA) with PE150 strategy at Personal Biotechnology Co., Ltd. (Shanghai, China). The instrument for measuring the quantity and quality of extracted DNA, along with the sequencing strategy, is described in Zhou et al. (2025).

### 2.6 Metagenomics analysis

Raw sequencing reads were processed to obtain quality-filtered reads for further analysis. First, sequencing adapters were removed from sequencing reads using Cutadapt (v1.2.1). Secondly, low quality reads were trimmed using a sliding-window algorithm in fastp. Megahit (v1.1.2) was used to assemble for each sample using the meta-large presetted parameters. The generated contigs (longer than 200bp) were then pooled together and clustered using mmseqs2 (Steinegger and SöDing, 2017) with “easy-linclust” mode, setting sequence identity threshold to 0.95 and covered residues of the shorter contig to 90%. The lowest common ancestor taxonomy of the non-redundant contigs was obtained by aligning them against the NCBI-nt database by mmseqs2 (Steinegger and SöDing, 2017) with “taxonomy” mode, and contigs assigned to Viridiplantae or Metazoa were dropped in the following analysis. MetaGeneMark was used to predict the genes in the contigs. CDS sequences of all samples were clustered by mmseqs2 (Steinegger and SöDing, 2017) with “easy-cluster” mode, setting protein sequence identity threshold to 0.90 and covered residues of the shorter contig to 90%. To assess the abundances of these genes, the high-quality reads from each sample were mapped onto the predicted gene sequences using salmon in the quasi-mapping-based mode with “--meta --minScoreFraction=0.55 “, and the TPM (transcripts per million) was used to normalize abundance values in metagenomes. Assembly and gene annotation, taxonomic filtering, and gene prediction and quantification reference Li et al. (2024).The functionality of the non-redundant genes were obtained by annotation using mmseqs2 (Steinegger and SöDing, 2017) with the “search” mode against the protein databases of KEGG.

### 2.7 Collection and extraction of root exudates

Fifteen plants were selected with consistent growth from different treatments, dug up, washed the roots with distilled water, placed in culture flasks filled with distilled water. After 24 hours of growth in the previously grown flowerpot, mixed solution from the culture flasks was collected to measure plant root exudates (5 plants per replicate, 3 replicates). Analysis of sample extracts used a UPLC-ESI-MS/MS system (UPLC, SHIMADZU Nexera X2, https://www.shimadzu.com.cn/; MS, Applied Biosystems 4500 Q TRAP, https://www.thermofisher.cn/cn/en/home/brands/applied-biosystems.html) (UPLC: Column, Agilent SB-C18 (1.8 µm, 2.1 mm × 100 mm). The mobile phase consisted of pure water containing 0.1% formic acid (solvent A) and acetonitrile (solvent B). Sample measurements were analyzed using a gradient program with starting conditions of 95% A and 5% B. A linear gradient injection was programmed from starting conditions of 5% A and 95% B in 9 min. The composition consisted of 5% A and 95% B infused continuously for 1 min. Following that, the 95% A and 5.0% B composition was modified over 1.1 min and maintained for 2.9 min. The flow rate was 0.35 mL per minute. The column oven temperature was set to 40°C. The injection volume was 4 μL. The effluent was bound to an ESI-triple quadrupolelinear ion trap (QTRAP)-MS alternatively Xu et al. (2023) for measuring root exudates.

### 2.8 Data analysis

All data analyses were performed using R (version 4.3.3). One-way ANOVA with Tukey’s test was used to evaluate the differences between the plant biomass and gene abundance among the four groups. Beta diversity was analyzed using principal coordinate analysis (PCA) based on Bray–Curtis distance metrics in the vegan package (Borcard et al., 2011). Differential metabolites were screened based on variable importance in projection (VIP) values extracted from OPLS-DA (Orthogonal partial least squares discriminant analysis) models, combined with fold change criteria in the MetaboAnalystR package (Thévenot et al., 2015). Pearson correlation analysis was used to assess the relationships between functional gene abundance and root exudates profiles.

## 3 Result

### 3.1 Effects of warming and nitrogen deposition on biomass and biomass allocation of *A. trifida*

**Figure 1:**
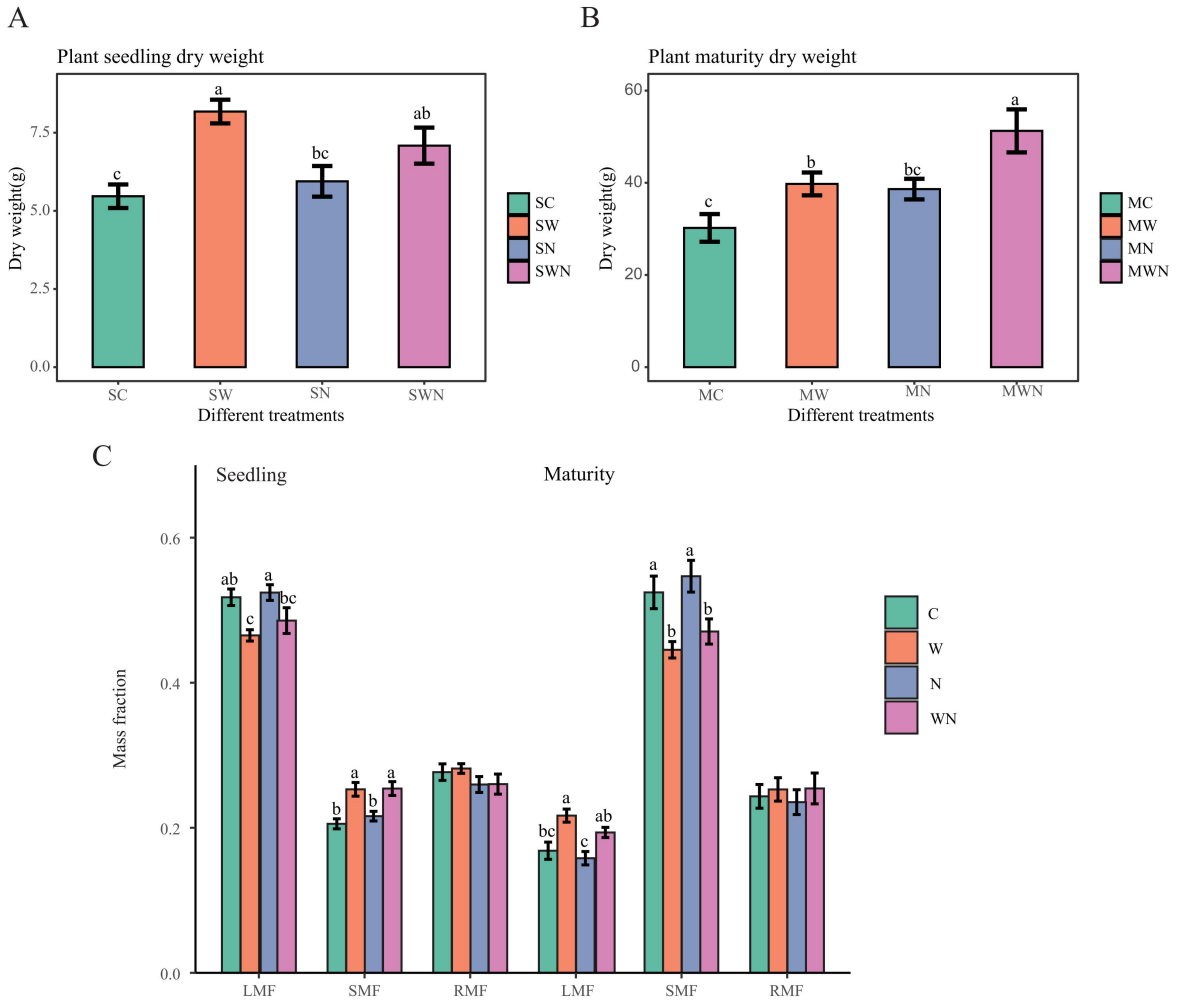
Effects of warming and nitrogen addition on biomass and biomass allocation of *A. trifida*. **(A)** Dry weight of plants at the seedling stage under four groups: control (SC), warming (SW), nitrogen addition (SN), and combined warming and nitrogen addition (SWN). **(B)** Dry weight of plants at the maturity stage under control (MC), warming (MW), nitrogen addition (MN), and combined warming and nitrogen addition (MWN). **(C)** Biomass allocation at seedling and maturity stages, represented by leaf mass fraction (LMF), stem mass fraction (SMF), and root mass fraction (RMF) under different treatments: control (C), warming (W), nitrogen addition (N), and warming plus nitrogen addition (WN).Different letters above bars indicate significant differences among treatments (*p* < 0.05).

Dry Biomass analysis of *A. trifida* across different treatments and growth stages revealed that warming significantly promoted plant growth. During the seedling stage, total dry biomass under the warming treatment (SW) was significantly higher than in the control (SC) and nitrogen addition (SN) treatments (**Figure 1A**). In the mature stage, biomass in the warming treatment (MW) was significantly greater than in the control (MC) (**Figure 1B**).

Nitrogen addition alone had no significant effect on total biomass at either growth stage compared to the control. However, the combined warming and nitrogen treatment (WN) significantly increased total biomass in both stages—exceeding that of SC and SN in the seedling stage and surpassing all other treatments in the mature stage. These results indicate that warming enhances biomass accumulation in *A. trifida*, and the combination of warming and nitrogen addition further amplifies this effect.

In terms of biomass allocation, the leaf mass fraction in SN was significantly higher than in SW, whereas the leaf mass fraction in MN was significantly lower than in MW. The stem mass fraction in SN was significantly lower than in SW, while in MN it was significantly higher than in MW and MWN. No significant differences in root mass fraction were observed among the four treatments (**Figure 1C**).

These results suggest that nitrogen addition during the seedling stage reduces belowground biomass allocation while increasing investment in leaves. In contrast, warming increases both root and leaf biomass allocation. Warming during the mature stage appears to promote leaf investment, likely to enhance light capture.

### 3.2 Impact of warming and nitrogen deposition on soil nutrient content

Analysis of the soil physicochemical properties under different treatments and growth stages revealed that soil nutrient levels and pH were highest under the combined warming and nitrogen addition treatment (SWN) during the seedling stage. In contrast, the nitrogen addition treatment during the mature stage (MN) showed the lowest soil nutrient content (**Table 1**).

**Table 1:** Physical and chemical properties of rhizosphere soil of *A. trifida* at different stages.

Treatment	N(g/kg)	SOC(g/kg)	P(g/kg)	K(g/kg)	pH
SC	0.46 ± 0.05b	5.74 ± 0.36b	0.25 ± 0.02bc	2.94 ± 0.06c	7.16 ± 0.00b
SW	0.44 ± 0.01b	5.29 ± 0.38c	0.27 ± 0.02b	2.96 ± 0.06c	7.28 ± 0.10b
SN	0.45 ± 0.02b	4.76 ± 0.44d	0.24 ± 0.02c	3.04 ± 0.05b	7.14 ± 0.08b
SWN	0.54 ± 0.03a	7.35 ± 0.16a	0.35 ± 0.02a	3.14 ± 0.05a	7.58 ± 0.08a
MC	0.42 ± 0.01A	6.49 ± 0.33A	0.18 ± 0.01A	2.76 ± 0.05B	7.20 ± 0.00A
MW	0.42 ± 0.02A	5.27 ± 0.80B	0.19 ± 0.01A	2.85 ± 0.06A	7.16 ± 0.03A
MN	0.33 ± 0.03C	3.32 ± 0.24C	0.16 ± 0.02B	2.54 ± 0.09C	7.18 ± 0.08A
MWN	0.38 ± 0.02B	5.32 ± 0.26B	0.18 ± 0.01AB	2.70 ± 0.05B	6.97 ± 0.05B

During the seedling stage, nitrogen addition alone did not significantly alter soil nutrient levels; however, the combination of warming and nitrogen addition significantly enhanced soil nutrient availability.

### 3.3 Soil microbial composition and diversity

**Figure 2:**
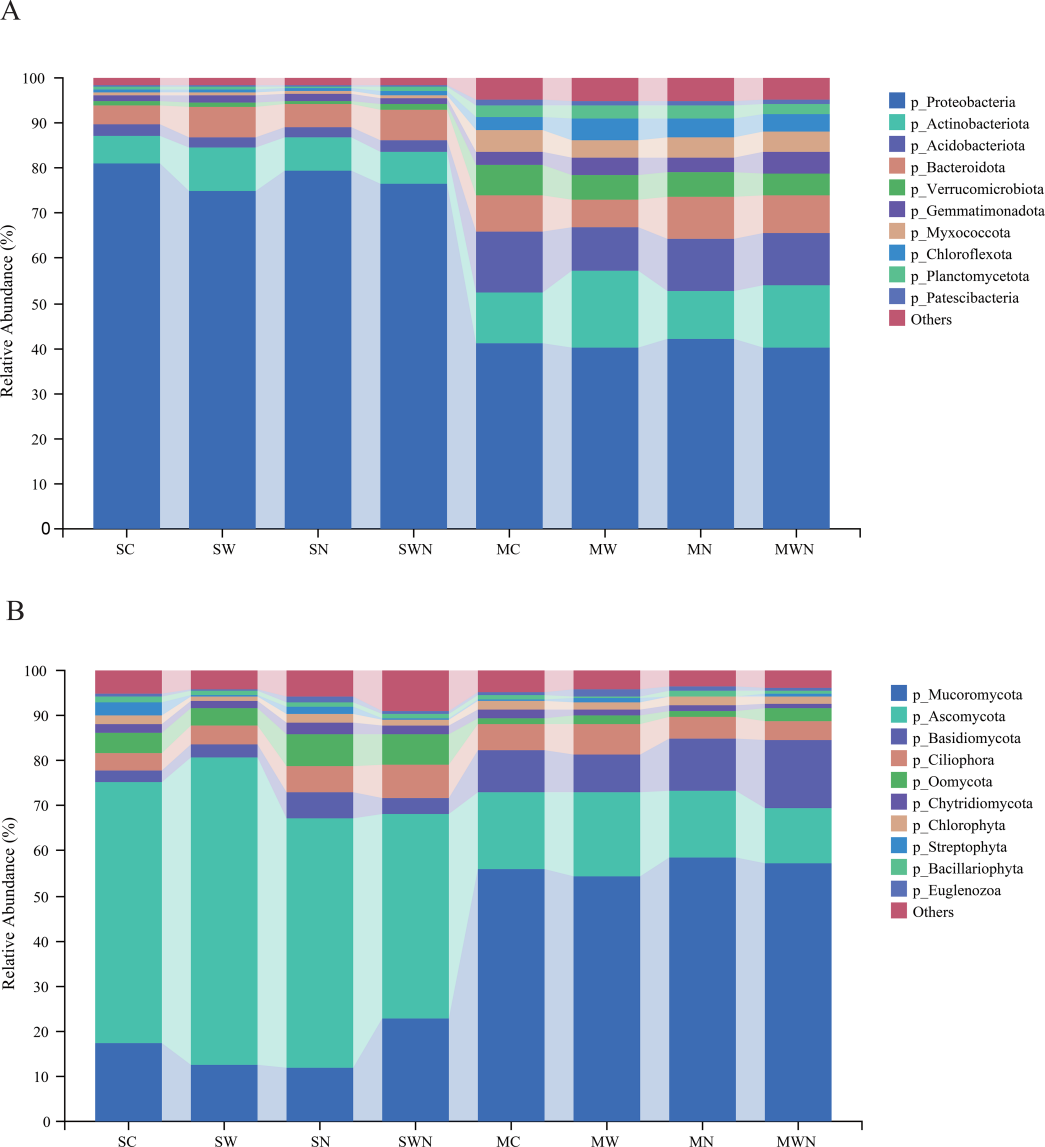
Effects of warming and nitrogen addition on the relative abundance of soil microbial taxa in the rhizosphere of *A. trifida*. **(A)** Relative abundance of dominant bacterial phyla under different groups at the seedling (SC, SW, SN, SWN) and maturity (MC, MW, MN, MWN) stages. **(B)** Relative abundance of dominant eukaryotic phyla under the same groups.

A total of 2,445,812,230 sequences were obtained through metagenomic sequencing. The number of each sample sequence is between 61,721,148 and 95,413,970. The abundance of Proteobacteria, Actinobacteriota, Bacteroidota, and Acidobacteriota in bacteria is relatively high, accounting for 80.9 ± 7.1%, 6.2 ± 1.8%, 2.5 ± 0.4%, and 4.3 ± 1.4% in SC, 74.9 ± 4.3%, 9.7 ± 3.2%, 2.1 ± 0.3%, and 6.9 ± 0.3% in SW, 79.4 ± 4.3%, 7.4 ± 2.8%, 2.3 ± 0.5%, and 5.1 ± 0.8% in SN, 76.6 ± 9.2%, 7.1 ± 3.5%, 2.4 ± 0.9%, and 6.8 ± 1.8% in SWN, 41 ± 1.6%, 11.2 ± 1.9%, 13.4 ± 1.2%, and 6.0 ± 1.9% in MC, 40.1 ± 6.0%, 17.1 ± 1.0%, 9.8 ± 1.3%, and 6.0 ± 3.6% in MW, 42.3 ± 1.6%, 10.4 ± 1.3%, 11.6 ± 0.7%, and 9.3 ± 1.2% in MN, 40.3 ± 2.1%, 13.8 ± 3.1%, 11.5 ± 1.5%, and 8.4 ± 2.3% in MWN. Although SW led to a reduced relative abundance of Proteobacteria, it did not affect their species richness. Warming significantly increased the abundance of Bacteroidota during the seedling stage compared to SC, and promoted higher levels of Actinobacteriota and Chloroflexota during the mature stage compared to MC (**Figure 2A**).

Among eukaryotic taxa, Ascomycota, Mucoromycota, and Basidiomycota were the most abundant. During the seedling stage, nitrogen addition (SN) significantly increased the abundance of Basidiomycota compared to the control (SC) (*p* < 0.05). In the mature stage, the nitrogen addition treatment (MN) showed the highest relative abundances of Basidiomycota, Chlorophyta, and Bacillariophyta (**Figure 2B**).

Alpha diversity analysis (Chao1, Shannon, Simpson indices) revealed no significant differences across treatments or growth stages, suggesting that the overall richness and evenness of microbial communities remained stable despite changes in composition (**Supplementary Table S1**).

### 3.4 Analysis of differentially abundant functional genes involved in carbon, nitrogen, and phosphorus cycling

Principal coordinate analysis (PCA), combined with similarity analysis (anosim), was used to evaluate the distribution of microbial communities carrying functional genes related to carbon, nitrogen, and phosphorus cycling under different treatments and growth stages.

**Figure 3:**
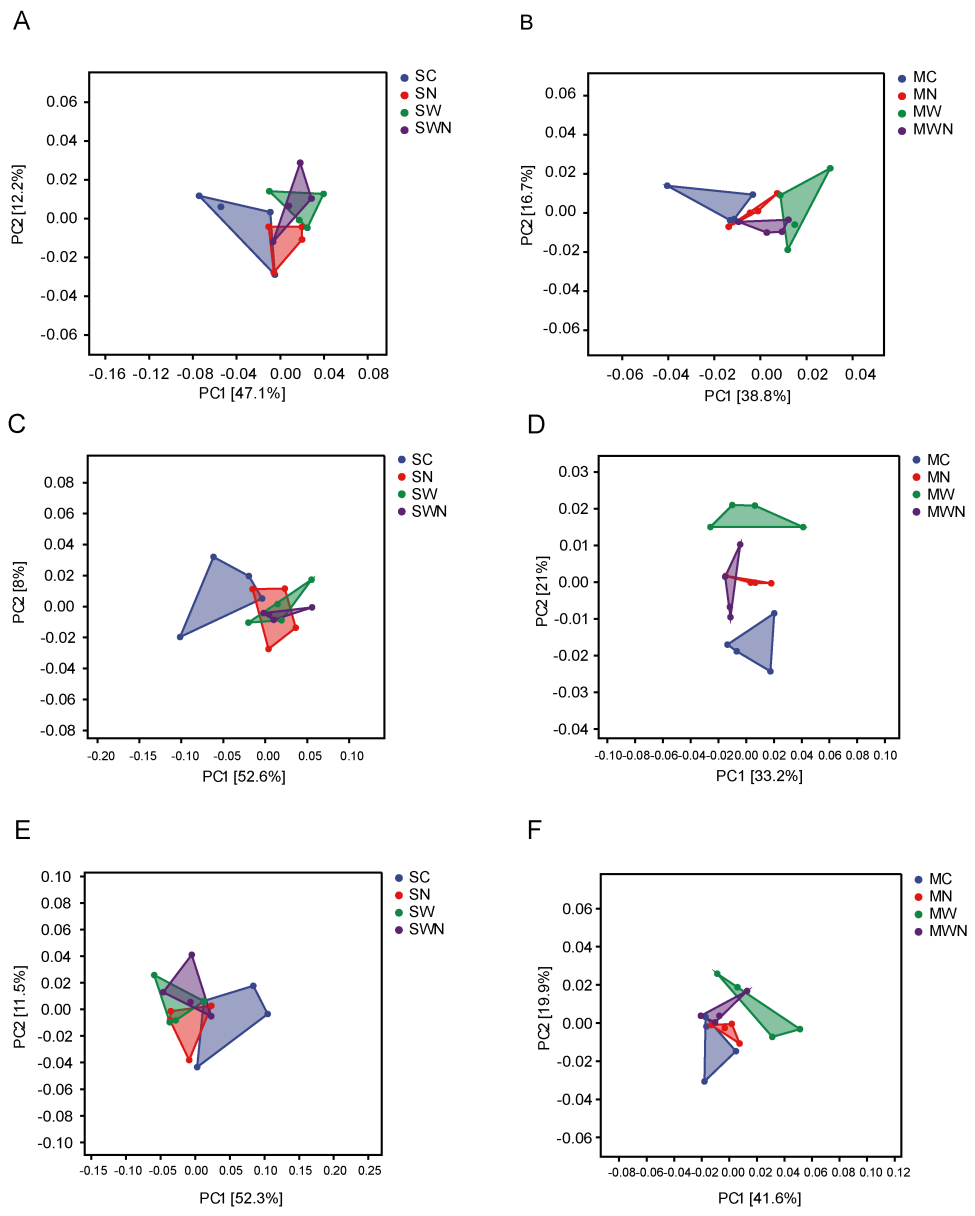
Principal coordinate analysis (PCA) of microbial communities associated with carbon, nitrogen, and phosphorus cycling genes in the rhizosphere of *A. trifida* under different groups and growth stages. **(A, B)** PCA plots of microbial functional genes related to carbon cycling. **(C, D)** PCA plots of microbial functional genes related to nitrogen cycling. **(E, F)** PCA plots of microbial functional genes related to phosphorus cycling.

The results revealed significant differences in the composition of microbial communities involved in C, N, and P cycling under warming and nitrogen addition treatments (*p* < 0.05), suggesting that climate change factors are key drivers influencing the structure of nutrient-cycling microbial communities (**Figure 3**).

**Figure 4:**
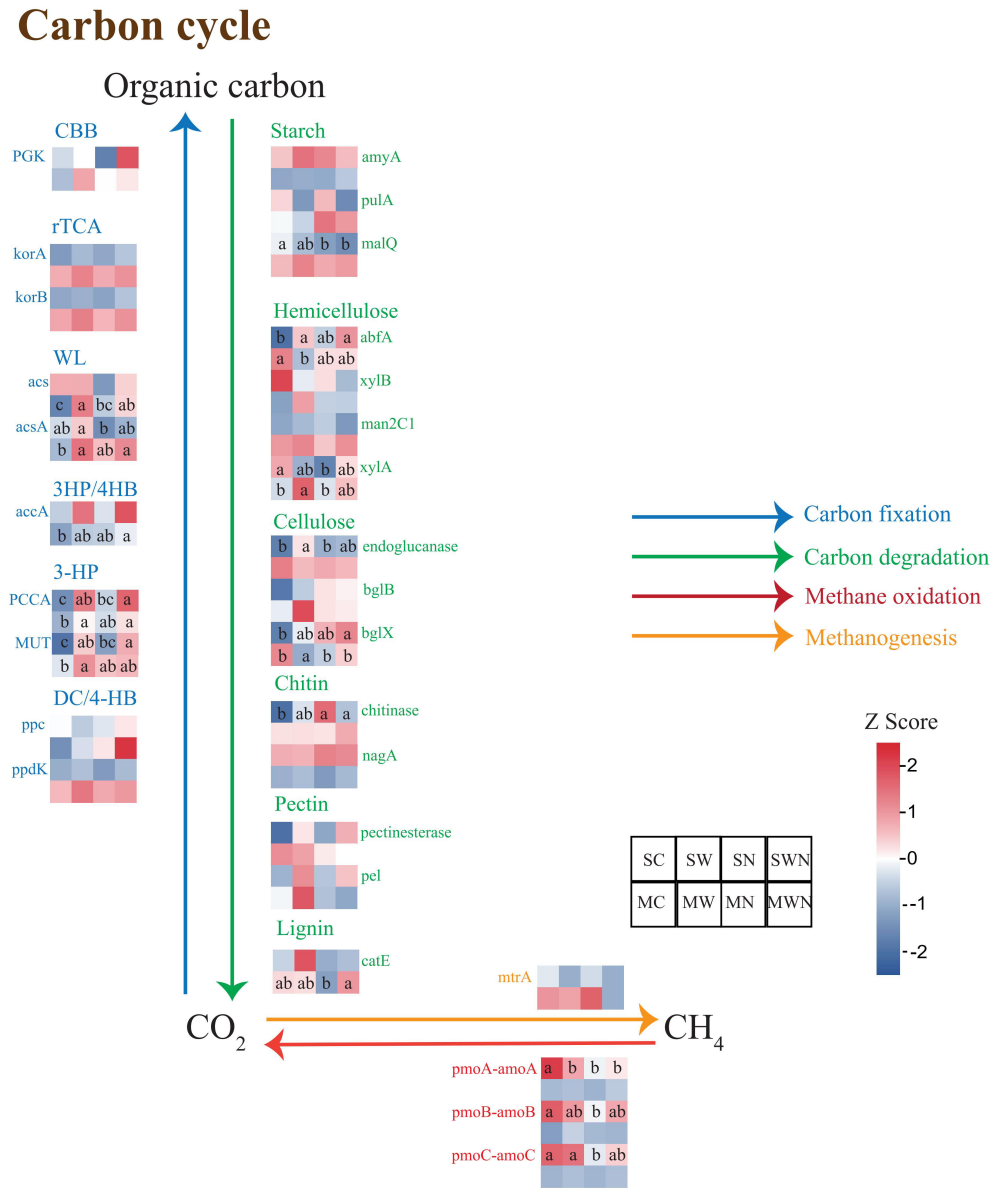
Heatmap analysis of differentially abundant functional genes involved in carbon cycling pathways in the rhizosphere of *A. trifida* under different treatments and growth stages. The figure illustrates the relative abundance of functional genes related to (i) carbon fixation (blue), (ii) carbon degradation of various organic polymers including starch, hemicellulose, cellulose, chitin, pectin, and lignin (green), (iii) methane oxidation (red), and (iv) methanogenesis (orange). Color gradients represent relative gene abundance, with red indicating higher abundance and blue indicating lower abundance.

A total of 29 carbon cycling-related functional genes were identified, including 10 associated with carbon fixation, 15 with carbon degradation, and 4 related to methane metabolism (**Figure 4**). Among the carbon fixation genes, the relative abundances of *ppcA* and *MUT* were significantly higher in the SW and SWN treatments compared to the control (SC). In the mature stage, the MW treatment significantly increased the abundances of *acs*, *acsA*, *PCCA*, and *MUT* relative to MC, while the MWN treatment significantly increased the abundances of *acs*, *acsA*, *accA*, *PCCA*, and *PPC* compared to MC. For carbon degradation genes, SW significantly elevated the abundance of *abfA* and endoglucanase compared to SC, while SWN increased the abundance of *abfA* and *bglX*. In the mature stage, MW significantly increased *xylA* and *bglX* abundances compared to MC. Regarding methane metabolism genes, nitrogen addition (SN) significantly reduced the abundances of *pmoA-amoA*, *pmoB-amoB*, and *pmoC-amoC* compared to the control (SC). These findings suggest that warming enhances carbon fixation and the decomposition of cellulose and hemicellulose in the rhizosphere soil of *A. trifida*, thereby promoting carbon cycling activity.

**Figure 5:**
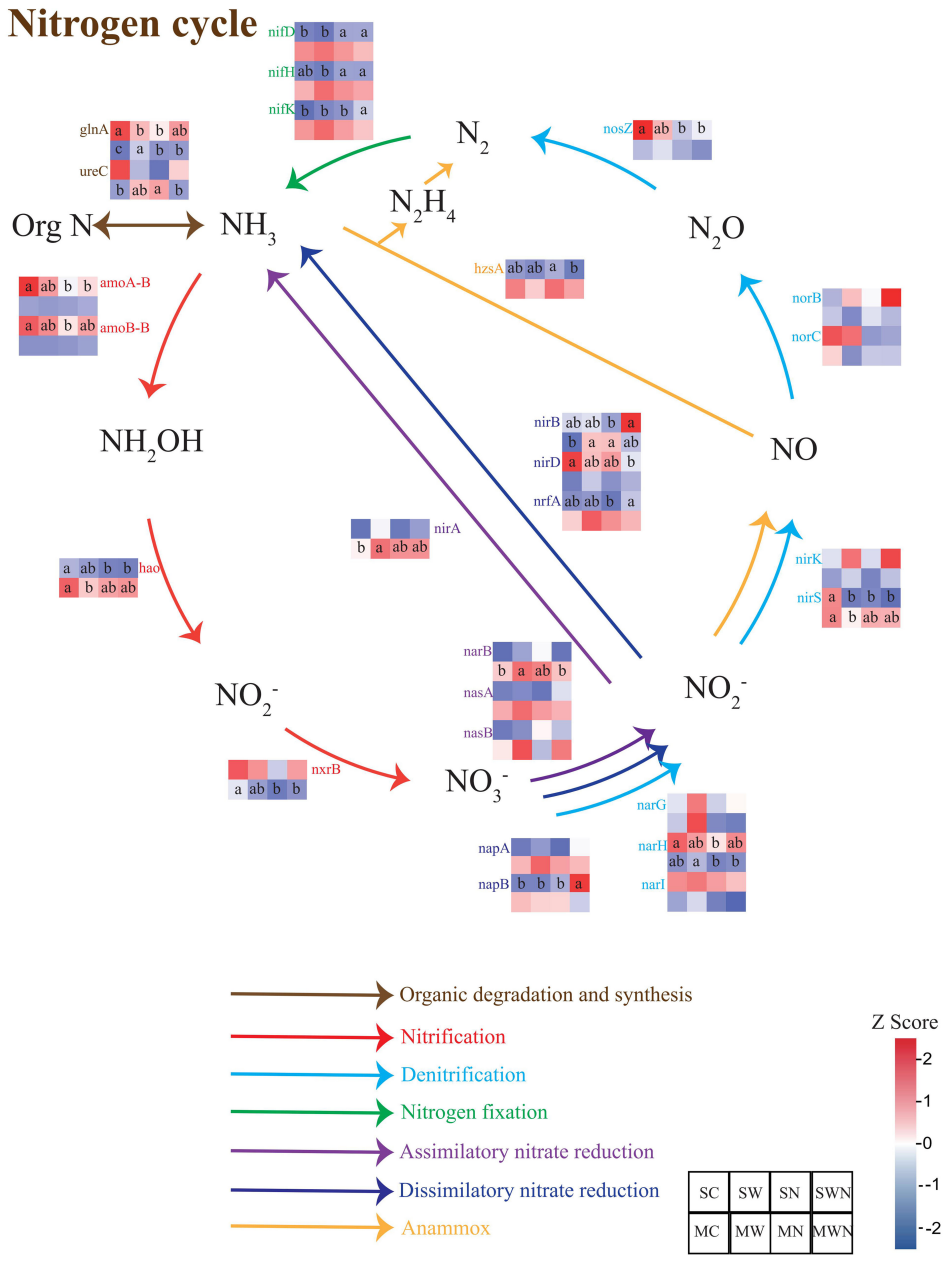
Heatmap visualization of differentially abundant functional genes involved in nitrogen cycling in the rhizosphere of *A. trifida* under different treatments and growth stages.

Analysis of nitrogen cycling functional gene abundance revealed that nitrogen addition during the seedling stage significantly reduced the relative abundance of *amoA-*B, *amoB-*B, *hao*, *narH*, *nirS*, and *nosZ* genes compared to the control (SC) (**Figure 5**). Similarly, in the combined warming and nitrogen addition treatment (SWN), the abundances of *amo*A*-*B, *hao*, *nirS*, and *nosZ* were also significantly lower than in SC, indicating that nitrogen addition notably inhibited both nitrification and denitrification processes. In contrast, nitrogen addition significantly increased the abundance of nitrogen fixation-related genes, such as *nifD*, compared to SC. Furthermore, compared to nitrogen addition alone (SN), the SWN treatment significantly elevated the abundances of *napB*, *nirB*, and *nrfA*, suggesting an enhancement of dissimilatory nitrate reduction under combined warming and nitrogen conditions. At the mature stage, the warming treatment (MW) significantly increased the abundances of *nirA* and *narB* relative to MC, indicating that warming promoted nitrogen assimilation.

**Figure 6:**
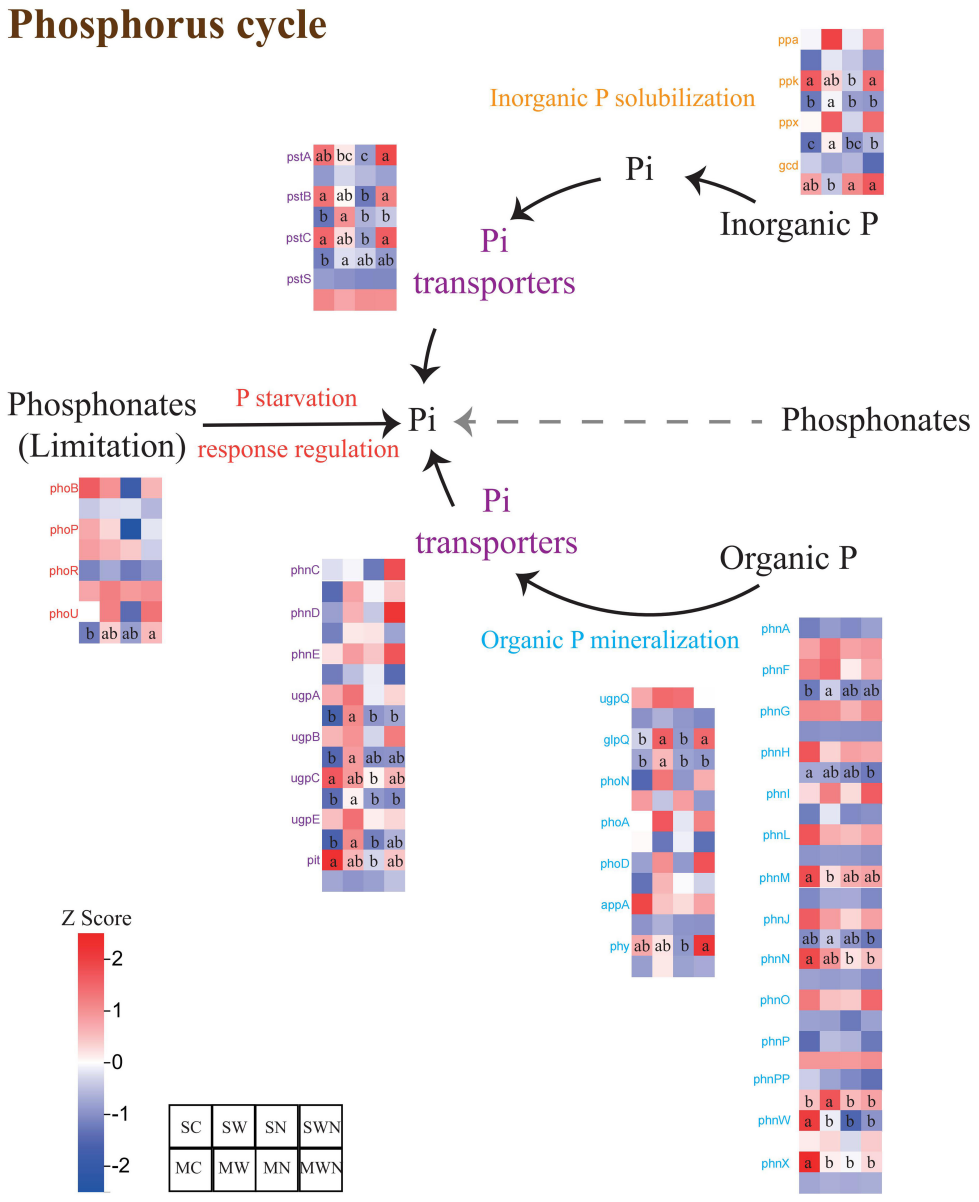
Heatmap of differentially abundant functional genes involved in phosphorus cycling in the rhizosphere of *A. trifida* under different treatments and growth stages.

Among all detected genes, those involved in microbial inorganic phosphorus solubilization, transport, and starvation response were the most abundant (**Figure 6**).

During the seedling stage, compared to the control (SC), only three genes—*phnM*, *phnW*, and *phnX*—showed significantly reduced abundance in the warming treatment (SW). In contrast, nine genes—*phnN*, *phnW*, *phnX*, *ppk*, *ugpC*, *pstA*, *pstB*, *pstC*, and *pit*—were significantly reduced in the nitrogen addition treatment (SN). In the mature stage, the warming treatment (MW) significantly increased the relative abundance of inorganic phosphorus solubilization genes (*ppk* and *ppx*), as well as several phosphorus mineralization genes, including *phnPP*, *phnF*, and *glpQ*, compared to the control (MC).

Additionally, most phosphorus transport genes—particularly *pstB*, *pstC*, and *ugpABCE*—were significantly upregulated under warming conditions.

### 3.5 Species contribution analysis of functional genes with differential carbon, nitrogen, and phosphorus cycling

**Figure 7:**
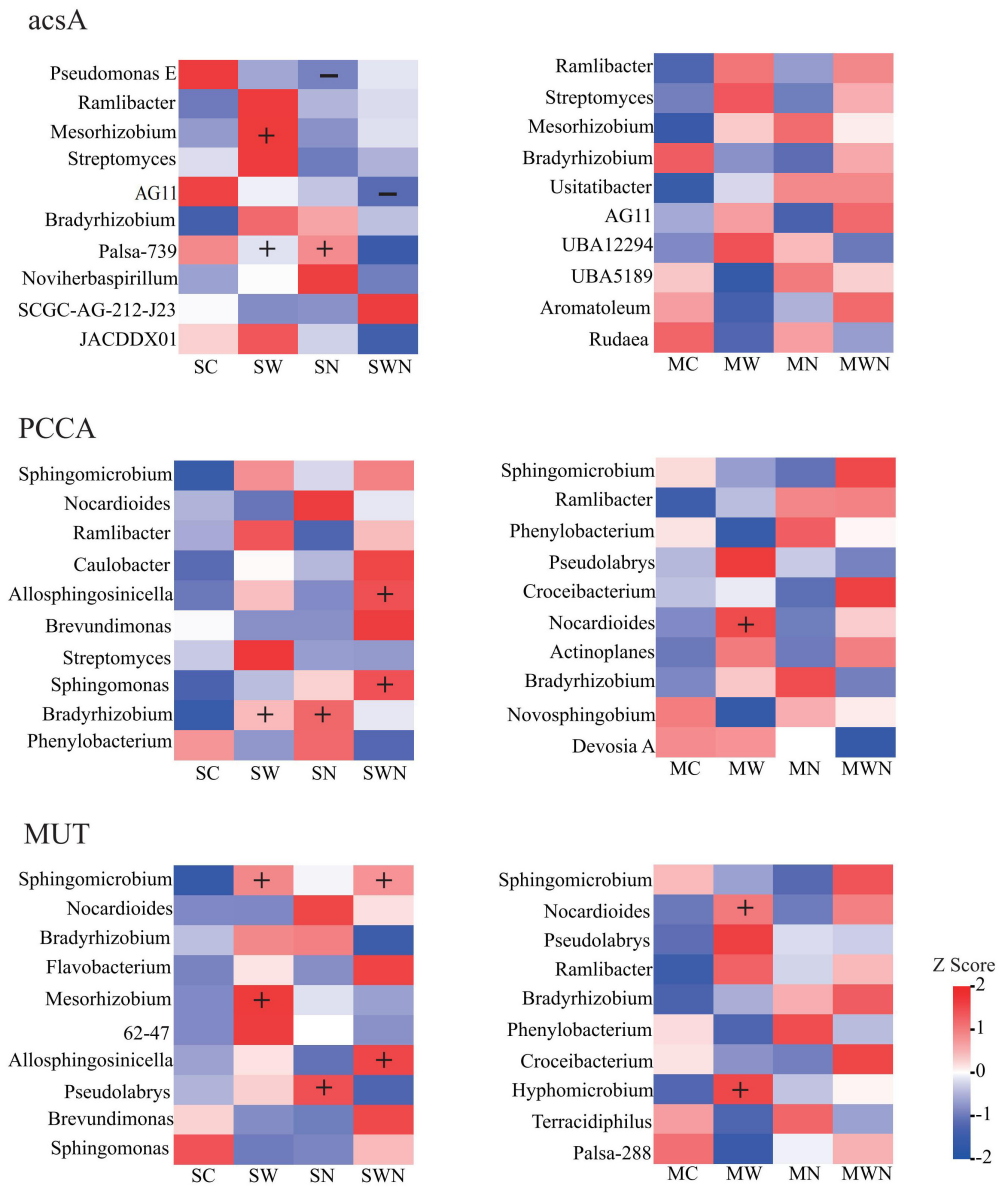
Heatmap of contribution of carbon fixed gene species.

Species contribution analysis was conducted on differentially expressed genes, and it was found that in the carbon cycle, SW increased the species abundance of *Mesorhizobium*, *Bradyrhizobium*, and *Sphingomicrobium* (**Supplementary Figure S1**). During the carbon fixation process, SW increased the abundance of the *Massilia* and *Sphingomicrobium* (**Figure 7**).

**Figure 8:**
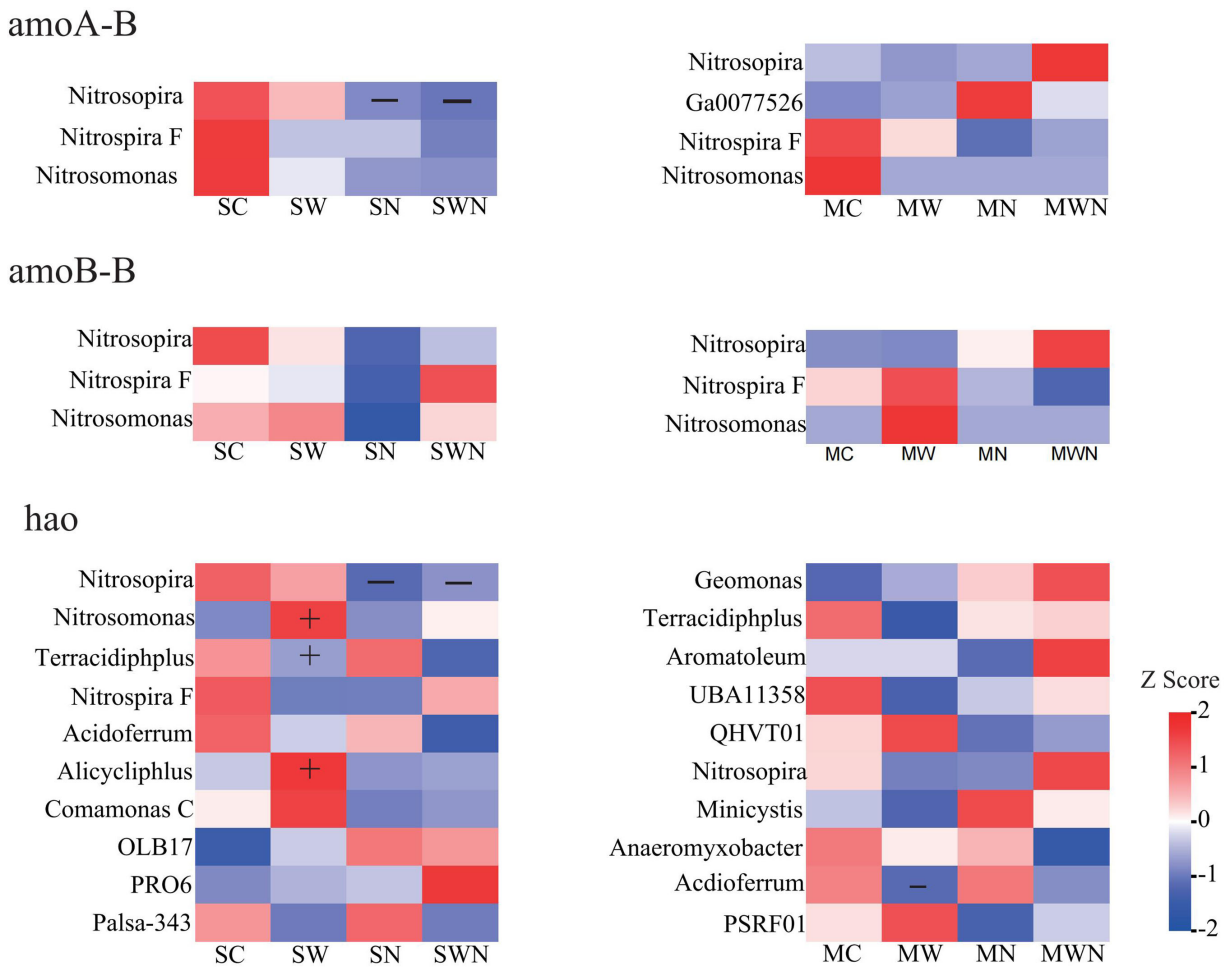
Heatmap of contribution of nitrification gene species.

During the nitrification process, SN reduced the abundance of *Nitrosospira* related bacteria, while SW increased the abundance of *Nitrosomonas*, *Terracidiphilus*, and *Alicycliphlus* related bacteria (**Figure 8**). During denitrification,SN reduced *Pseudomonas* and increased *Azospirillum* abundance, while MN increased *Ramlibacter* abundance. SW increased the abundance of *Sphingomicrobium* and *Massilia*, while reducing the abundance of *Pseudomonas* and *Trinickia*. MW reduced the abundance of *Rhizomicrobium* (**Supplementary Figure S2***)*. And during nitrogen fixation, SN increased the abundance of *Azospirillum* (**Supplementary Figure S3**).

**Figure 9:**
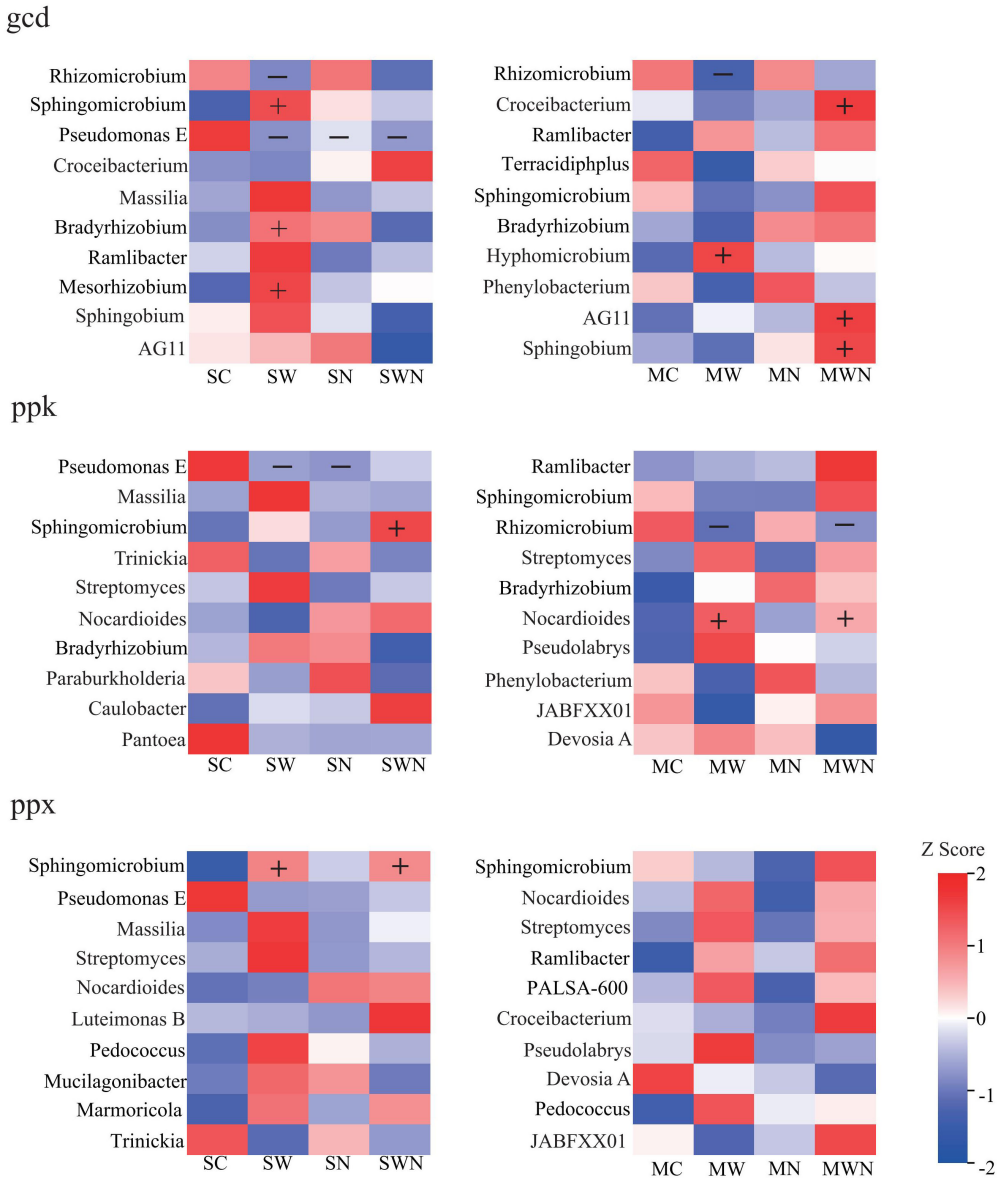
Heatmap of contribution of inorganic phosphorus dissolution gene species.

During the process of inorganic phosphorus dissolution, SW increased the relative abundance of *Mesorhizobium*, *Sphingomicrobium*, and *Bradyrhizobium*, while reducing the relative abundance of *Rhizomicrobium*, *Trinickia*, and *Pseudomonas*. MW had increased the relative abundance of *Nocardioides* (**Figure 9**). During the process of phosphorus transport, SW led to an increase in *Sphingomicrobium*, while reducing the growth of *Pseudomonas* (**Supplementary Figure S4**). Compared with the process of organic phosphorus mineralization, SW increased the relative abundance of *Streptomyces* and *Massilia*, and MW also increased the relative abundance of *Nocardioides* (**Supplementary Figure S5**).

### 3.6 Different types and quantities of root exudates

A total of 684 metabolites were detected, including 28 amino acids and derivatives, 57 nucleotides and derivatives, 89 organic acids, 138 lipids, 148 phenolic acids, 23 flavonoids, 40 lignans and coumarins, 28 alkaloids, and 16 terpenoids.

**Figure 10:**
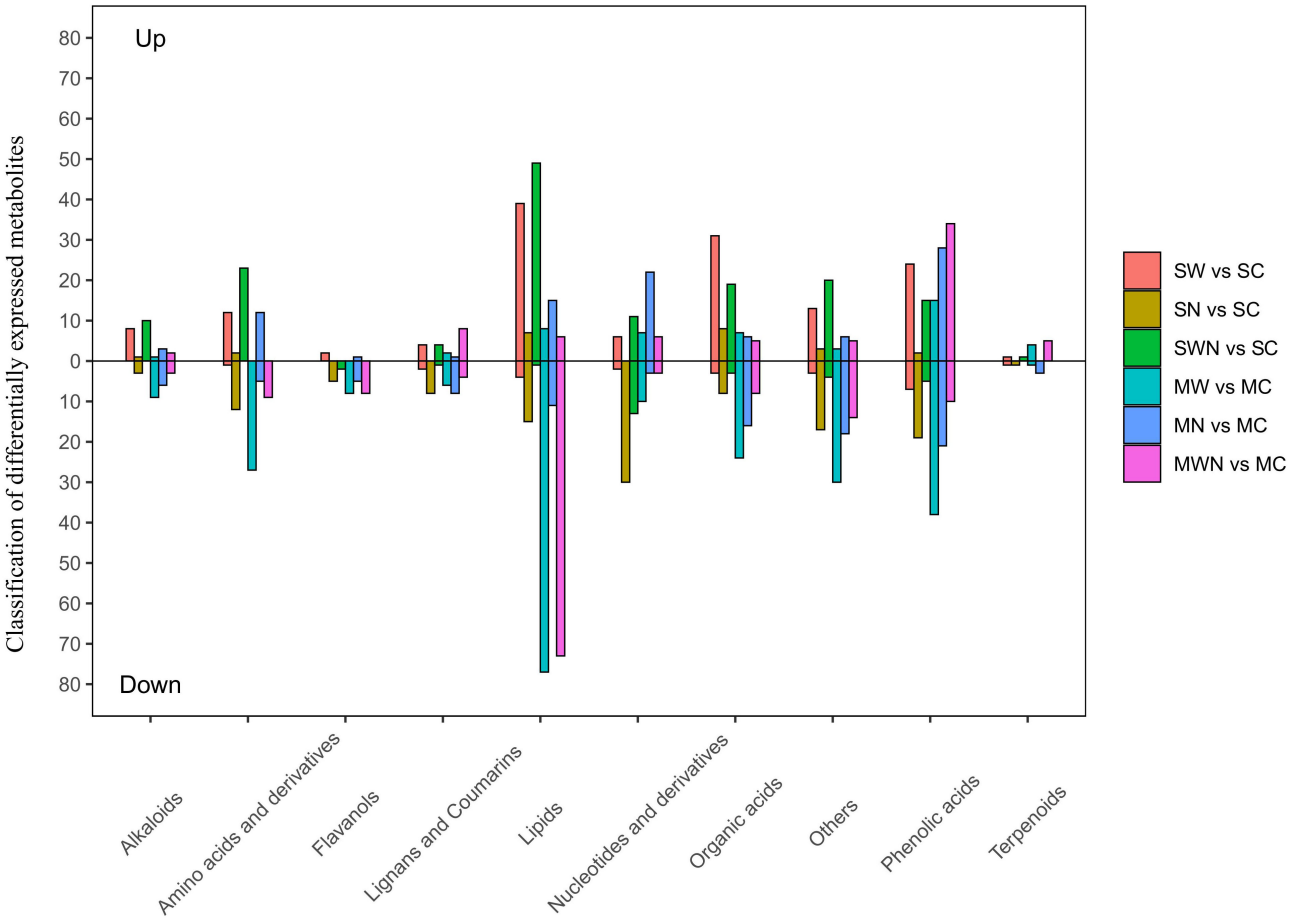
Classification of differentially expressed metabolites in *A. trifida* rhizosphere under warming and nitrogen treatments at different growth stages.

We choose compounds with fold change ≥ 2, fold change ≤ 0.5, and VIP ≥ 1 as differential metabolites. There are differences in the composition of metabolites in different treatments at different stages, with the number of upregulated compounds being higher than the number of downregulated compounds, indicating that the warming and nitrogen addition at different stages may activate key physiological metabolites activity. Further classify and compare the different metabolites produced by different treatments. Compared with SC, SW up regulated 14 amino acids, 38 lipids, 34 organic acids, and 21 phenolic acids, while down regulated 1 amino acid, 4 lipids, 3 organic acids, and 7 phenolic acids; In SN, 1 alkaloid, 8 organic acids, and 2 phenolic acids were up regulated, 3 alkaloids, 8 organic acids, and 19 phenolic acids were down regulated; In SWN, 23 amino acids, 49 lipids, 19 organic acids, and 15 phenolic acids were up regulated, while 1 lipid, 3 organic acids, and 5 phenolic acids were down regulated. Compared with MC, 8 lipids, 7 organic acids, and 15 phenolic acids were up regulated in MW, while 27 amino acids, 77 lipids, 24 organic acids, and 38 phenolic acids were down regulated; In MN, there were 6 organic acids, 3 alkaloids, and 28 phenolic acids up regulated, 6 alkaloids, 16 organic acids, and 21 phenolic acids down regulated; In MWN, 6 lipids, 5 organic acids, and 34 phenolic acids were up regulated, while 9 amino acids, 73 lipids, 8 organic acids, and 10 phenolic acids were down regulated (**Figure 10**).

### 3.7 Correlation analysis between chemical composition of root exudates and soil nutrient cycling functional genes

**Figure 11:**
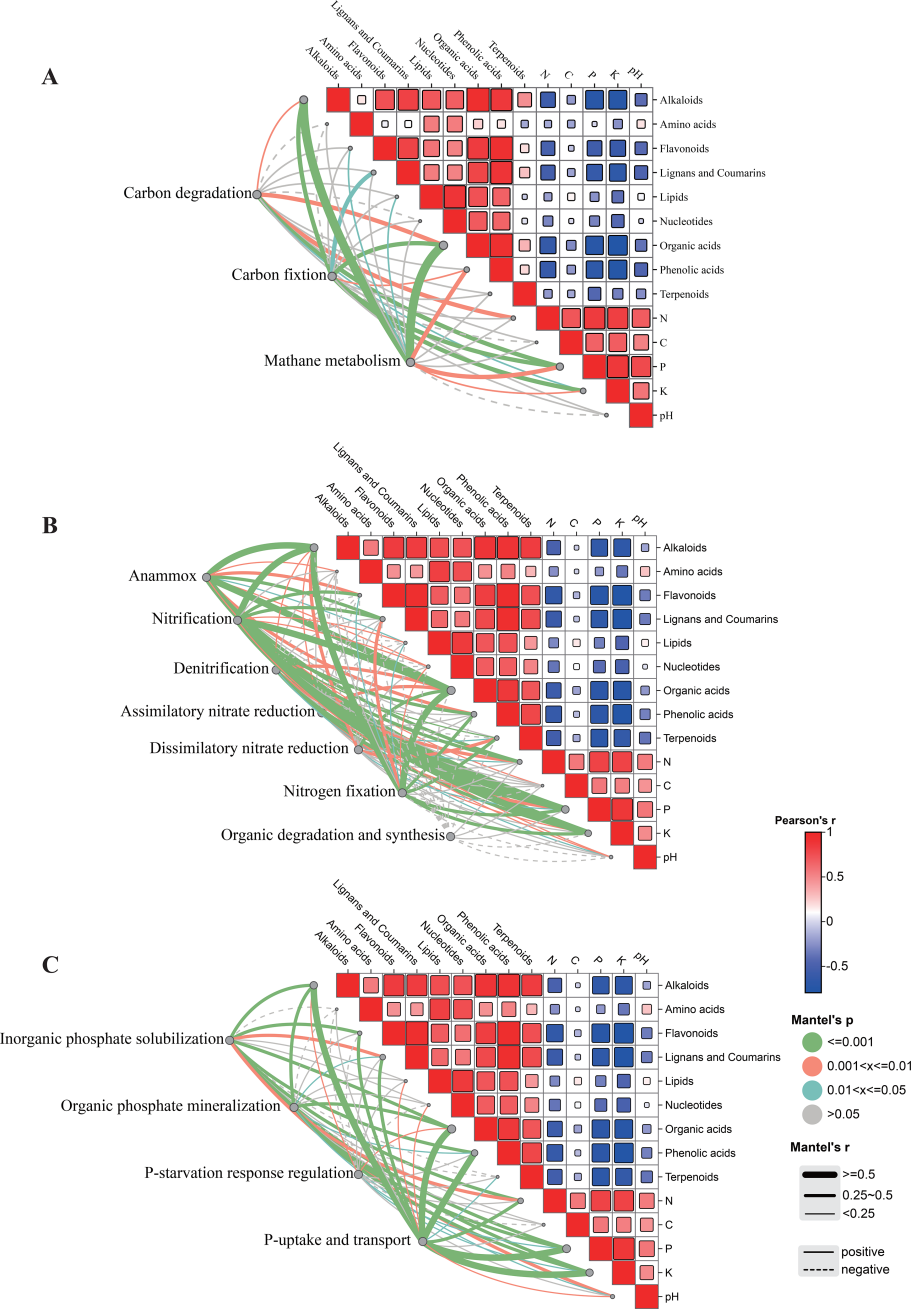
Correlation analysis between root exudate metabolites and functional genes involved in **(A)** carbon cycling, **(B)** nitrogen cycling, and **(C)** phosphorus cycling in the rhizosphere of *A. trifida*.

Correlation matrix analysis of environmental factors revealed that, within the carbon cycle, carbon degradation genes showed a weak but significant positive correlation with organic acids (*r* = 0.39, *p* < 0.05). Similarly, carbon fixation genes were weakly correlated with organic acids (*r* = 0.39, *p* < 0.05), alkaloids (*r* = 0.36, *p* < 0.05), and coumarins (*r* = 0.28, *p* < 0.05) (**Figure 11A**).

In the nitrogen cycle, functional genes associated with anaerobic ammonia oxidation were significantly correlated with alkaloids (*r* = 0.52, *p*< 0.05) and organic acids (*r* = 0.57, *p* < 0.05). Genes involved in nitrification and nitrogen fixation also showed significant correlations with alkaloid and organic acid concentrations (**Figure 11B**).

In the phosphorus cycle, genes related to phosphorus absorption and transport were strongly and significantly correlated with alkaloids (*r*= 0.66, *p* < 0.05), organic acids (*r*= 0.71, *p* < 0.05), and phenolic acids (*r*= 0.55, *p* < 0.05) (**Figure 11C**).

**Figure 12:**
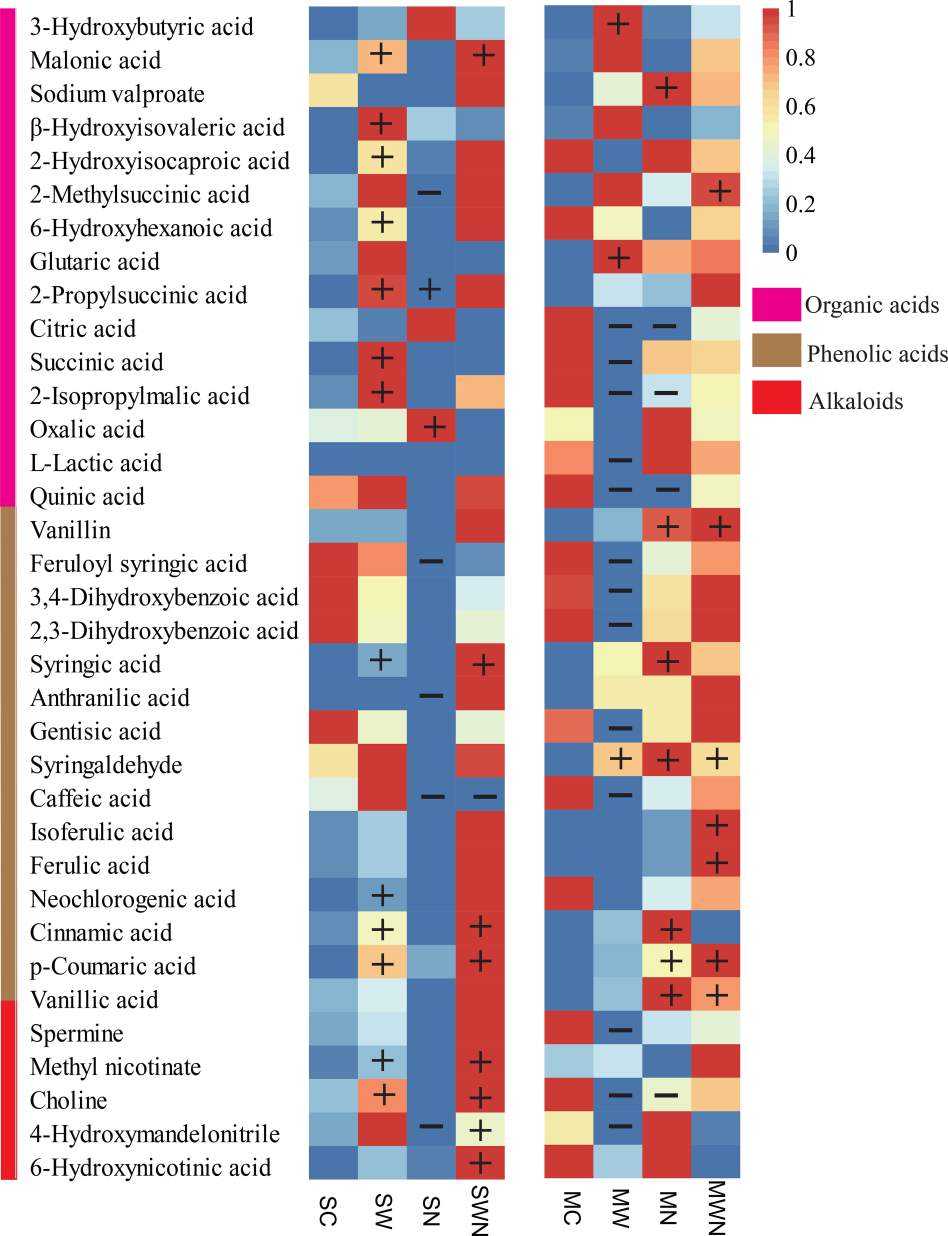
Heatmap of differentially accumulated metabolites in the rhizosphere of *A. trifida* under warming and nitrogen addition treatments. The plus (+) and minus (−) symbols indicate metabolites that were significantly different from the control group, fold change ≥ 2 or ≤ 0.5 and a VIP score ≥ 1.

Analysis of differential metabolites revealed distinct changes in organic acids, phenolic acids, and alkaloids under different treatments. In the organic acid category, the warming treatment (SW) increased the concentrations of malonic acid, β-hydroxyisovaleric acid, 2-hydroxyisocaproic acid, 6-hydroxyhexanoic acid, 2-propylsuccinic acid, succinic acid, and citric acid. In contrast, nitrogen addition (SN) reduced the levels of malonic acid and 2-methylsuccinic acid. For phenolic acids, SW treatment led to elevated levels of syringic acid, neochlorogenic acid, cinnamic acid, and p-coumaric acid, whereas SN treatment resulted in reduced levels of feruloyl syringic acid, anthranilic acid, and caffeic acid (**Figure 12**).

In the alkaloid group, SW increased the concentrations of methyl nicotinate and choline. Additionally, the combined warming and nitrogen treatment (SWN) significantly increased the levels of 4-hydroxymandelonitrile and 6-hydroxynicotinic acid.

## 4 Discussion

Rising temperatures typically have a positive effect on biomass accumulation in invasive plant species. Our experimental results show that warming significantly increases the biomass of *A. trifida*, whereas nitrogen addition alone does not produce a significant effect. In addition, both warming and nitrogen deposition influence biomass allocation patterns in invasive plants. For instance, the increased allocation to leaves observed in the nitrogen addition treatment (SN) may be due to reduced allocation to belowground tissues. Although the leaf mass fraction in the SW and SWN treatments was lower than in SN, the absolute leaf biomass was highest in the SW group. These findings suggest that warming (+2°C) enhances the invasiveness of *A. trifida*, likely by promoting aboveground growth, thereby increasing its potential to expand its geographic range.

A meta-analysis has shown that warming typically does not reduce microbial alpha diversity (Zhou et al., 2020). Guo et al. (2022) reported that five years of global warming altered the seasonal dynamics of soil microbial communities in grassland ecosystems, significantly increasing the relative abundance of Actinobacteria and Chloroflexi. In our study, the mature-stage abundance of Actinobacteria and Chloroflexi was also significantly higher under warming compared to the control (*p* < 0.05). These two phyla are often considered key microbial indicators sensitive to warming, as supported by several studies (Oliverio et al., 2017; Reimer et al., 2022; Rodrigues et al., 2013). These organisms tend to be eutrophic, characterized by rapid growth and preference for nutrient-rich soils. Enhanced availability of labile carbon under warming may therefore stimulate the proliferation of α-Proteobacteria. (Zhao et al., 2024). Compared with warming, nitrogen addition appeared to have a limited effect on microbial abundance in our study, possibly due to the relatively short duration of nitrogen application (Zhang et al., 2018). Although neither warming nor nitrogen deposition significantly altered the alpha diversity indices of the rhizosphere microbiota in *A. trifida*, both treatments significantly affected the composition and structure of microbial communities involved in nutrient cycling.

Integrating microbial community structure and function into plant invasion research is essential for understanding how nutrient cycling processes are modified during invasion. After confirming that the invasion of *A. trifida* alters both microbial community structure and function, we examined functional genes involved in carbon, nitrogen, and phosphorus cycling. We found that warming significantly increased the abundance of carbon fixation genes, particularly those associated with the reductive acetyl-CoA pathway (Wood–Ljungdahl or WL cycle), the 3-hydroxypropionate/4-hydroxybutyrate (3HP/4HB) cycle, and the 3-hydroxypropionate (3HP) bicycle. The WL cycle is a CO_2_ fixation pathway identified in hydrogen-utilizing autotrophic anaerobic bacteria, such as acetogenic, sulfate-reducing, and methanogenic bacteria. Unlike the reductive pentose phosphate pathway, which directly fixes CO_2_, the 3HP cycle fixes inorganic carbon in the form of HCO_3_^−^. The 3HP/4HB cycle is considered the most energy-efficient aerobic carbon fixation pathway and is particularly well adapted to nutrient-limited environments (Alfreider et al., 2018). Our findings indicate that microbial carbon metabolism is highly sensitive to temperature, and warming enhances the carbon fixation potential of microbial communities in soils invaded by *A. trifida*. In contrast, short-term nitrogen deposition did not lead to significant changes in microbial carbon cycling.

Following nitrogen addition, the abundance of soil nitrification-related genes decreased, possibly due to elevated concentrations of ammonium nitrogen in the soil. High levels of ammonium can be toxic to nitrifying bacteria, leading to their inhibition and a subsequent decline in nitrification gene abundance (Cua and Stein, 2011). Additionally, ammonia-oxidizing bacteria typically function optimally at temperatures between 20 °C and 30 °C. Warming may suppress their activity, without necessarily increasing the abundance of nitrification-related genes (Hallam et al., 2006; Dai et al., 2020). Nitrification and denitrification are the primary microbial processes responsible for mineral nitrogen loss in terrestrial ecosystems. Nitrification converts ammonium into nitrate, which can then be lost through leaching or transformed into nitrogen gas (N_2_) via denitrification—ultimately reducing nitrogen availability in plant–soil systems (Moreau et al., 2019). Our findings indicate that nitrogen addition inhibits both nitrification and denitrification, potentially reducing nitrogen losses from the soil. Notably, the abundance of functional genes involved in these processes did not significantly change under the combined warming and nitrogen treatments. In contrast, nitrogen addition increased the abundance of nitrogen fixation-related genes, which may enhance *A. trifida’s* ability to utilize soil nutrients and maintain growth under changing environmental conditions.

Phosphorus is an essential nutrient for the growth of all living organisms and is one of the most limiting elements in terrestrial ecosystems (George et al., 2016). Global warming accelerates the decomposition of organic matter, providing additional energy sources for soil microorganisms (Dungait et al., 2012). This stimulation leads to increased microbial and enzymatic activity, shifts in microbial metabolism, and changes in community composition, collectively accelerating phosphorus cycling processes—including the solubilization of inorganic phosphorus and mineralization of organic phosphorus (Bai et al., 2013; Lladó et al., 2017).

The *ppk* gene encodes polyphosphate kinase, which catalyzes the polymerization of phosphate monomers into polyphosphate chains. *ppa* encodes inorganic pyrophosphatase, and *ppx* encodes exopolyphosphatase, both of which are involved in hydrolyzing polyphosphates into plant-available phosphate (Wu et al., 2022). Additionally, the *pstS*, *pstB*, *pstA*, and *pstC* genes encode components of the high-affinity phosphate transporter (*PstSBAC*) system, which promotes phosphorus uptake under low-availability conditions.

The high abundance of *ppk* and *pstSBAC* genes observed in the rhizosphere of warming-treated *A. trifida* suggests an enhanced microbial capacity for phosphorus assimilation. Furthermore, warming increased the abundance of *ugpA*, *ugpB*, *ugpC*, and *ugpE*—genes encoding components of the sn-glycerol-3-phosphate (G3P) transport system, including permease proteins, substrate-binding proteins, and ATP-binding proteins.

In contrast, nitrogen addition appeared to have a negative effect on phosphorus cycling. Nitrogen input can lower soil pH, inhibit microbial growth, and alter microbial community composition, collectively reducing the microbial capacity for phosphorus solubilization.

These findings suggest that under future climate change, the impact of warming on phosphorus cycling—particularly in the context of invasive plant species—deserves greater attention than nitrogen deposition alone. In the analysis of the contribution of microbial taxa to functional gene abundance, we found that warming generally increased the relative abundance of *Sphingomicrobium*, *Massilia*, and *Nocardioides*, while decreasing *Pseudomonas*, *Trinickia*, and *Rhizomicrobium*.

*Pseudomonas* is known for its ability to reduce nitrate to N_2_ under anaerobic conditions and possesses strong phosphorus-solubilizing capabilities (Liu et al., 2024). *Rhizomicrobium* enhances plant phosphorus uptake by secreting organic acids and phosphatases that convert insoluble phosphorus into plant-available forms such as H_2_PO_4_^−^ and HPO_4_^2−^, thereby promoting plant growth (Sindhu et al., 2014). These beneficial bacterial genera tend to have relatively high environmental requirements, and their abundance may decline under suboptimal conditions. In contrast, *Sphingomicrobium* is characterized by a high metabolic capacity and multifunctional physiological traits, allowing it to effectively compete for soil resources and accumulate under warming conditions. Some *Massilia* strains possess nitrate reductase activity and contribute to soil carbon and nitrogen cycling (Hrynkiewicz et al., 2010). Additionally, research on maize rhizosphere microorganisms has shown that *Massilia* species can utilize photosynthetic products secreted by fungi, facilitating the mineralization and transformation of organic phosphorus in the soil (Wang et al., 2016).

The enrichment of *Sphingomicrobium*, *Massilia*, and *Nocardioides* under warming suggests that *A. trifida* may enhance soil nitrogen and phosphorus cycling through microbial shifts. In contrast, nitrogen addition alone had relatively limited effects on microbial community composition. Different types of organic acids function both as nutrient sources and signaling molecules for microorganisms during the colonization of beneficial rhizobacteria (Zhuang et al., 2013), and they represent a key mechanism by which plants enhance phosphorus uptake from the soil. In our experiment, warming during the seedling stage increased the secretion of several organic acids in *A. trifida*, including malonic acid, β-hydroxyisovaleric acid, 2-hydroxyisocaproic acid, glutaric acid, 2-propylsuccinic acid, and succinic acid, thereby influencing phosphorus absorption and transport.

In addition, previous studies have shown that invasive plants release phenolic acids and alkaloids with allelopathic properties, which can inhibit the growth of surrounding native plants. For example, chlorogenic acid, ferulic acid, and syringic acid have been shown to suppress bacterial growth and soil enzyme activity, ultimately inhibiting seedling development in native plant species (Chomel et al., 2016; Harrison et al., 2003; John and Sarada, 2012).

With respect to alkaloids, our results indicate that warming during the seedling stage significantly increased the concentrations of methyl nicotinate and choline, while combined warming and nitrogen addition led to increased levels of 4-hydroxymandelonitrile and 6-hydroxynicotinic acid. Choline is an organic base capable of forming complexes with soil cations, thereby improving the solubility and mobility of nutrient ions, which enhances nutrient uptake and promotes plant growth. It also acts as a natural antioxidant, mitigating oxidative stress and protecting plant cells. Furthermore, choline and its derivative phosphatidylcholine are essential components of eukaryotic cell membranes and play a role in facilitating rhizobial colonization (Gao et al., 2017). The exogenous application of 6-hydroxynicotinic acid has been shown to significantly enhance plant height, leaf length, root activity, nitrogen and phosphorus content, and total biomass in natural soils (Jiang et al., 2024). Our findings reveal that organic acid and alkaloid levels increased significantly under warming conditions during the seedling stage, whereas phenolic acid concentrations increased notably under nitrogen addition during the mature stage. These results suggest that *A. trifida* can dynamically adjust the types and quantities of root-secreted compounds in response to environmental conditions, thereby modifying soil nutrient cycling and gaining a competitive advantage over native vegetation.

Low-phosphorus cultivated land is widely distributed across China, with notable regional differences. North China, Southwest China, and Northwest China are the primary areas characterized by phosphorus deficiency. From the 1980s to the 2010s, the total available phosphorus in forests, grasslands, paddy fields, and arid ecosystems increased at a rate of 0.13 kg P ha^−1^ year^−1^. However, total soil phosphorus storage declined significantly during the same period, by approximately 4.5 kg P ha^−1^ year^−1^ (Song et al., 2024).Recent research has shown that invasive plants can successfully colonize phosphorus-deficient environments by releasing root carboxylates to mobilize soil phosphorus (Tang et al., 2025). Our findings suggest that under global warming, the spread of *A. trifida* may be further exacerbated in the nutrient-poor soils of southern China. Therefore, phosphorus management represents a promising strategy for controlling invasive species. Our findings improve the understanding of how invasive plants adapts to phosphorus-limited conditions, thereby informing the development of potential management practices.

## 5 Conclusion

Using targeted metabolomics and soil microbial metagenomic analysis, we found that future global warming promotes biomass accumulation in *A. trifida*, while nitrogen deposition plays a supportive role primarily during the later stages of plant development. *A. trifida* enhances soil carbon, nitrogen, and phosphorus cycling by enriching beneficial microbial taxa such as *Sphingomicrobium*, *Massilia*, and *Nocardioides*, likely through increased secretion of organic acids, phenolic compounds, and alkaloids in root exudates. Among all nutrient cycles, the most pronounced increase was observed in the abundance of functional genes related to phosphorus cycling. While nitrogen addition supports plant growth at later stages, warming exerts a more consistent and pronounced positive effect throughout the growth cycle. These findings suggest that warming may facilitate the successful invasion of *A. trifida* in phosphorus-limited environments, offering new insights into the microbial and chemical mechanisms underlying invasive plant success under climate change.

## Data Availability

The datasets presented in this study can be found in online repositories. The names of the repository/repositories and accession number(s) can be found in the article/**Supplementary Material**.
