# Supplementary material for: Warming outweighs nitrogen deposition in shaping rhizosphere microbial structure involved in carbon, nitrogen, and phosphorus cycling in Ambrosia trifida
Source: Front Plant Sci. 2026 Feb 10;17:1686326. doi: 10.3389/fpls.2026.1686326 (PMC12929440; doi:10.3389/fpls.2026.1686326)
Supplement: Supplementary file 1 [file DataSheet1.doc]

Table S1 *A*. *trifida* root associated microbial diversity index

|  |  | Chao1 | Shannon | Simpson | Pielou_e |
| --- | --- | --- | --- | --- | --- |
| Bacteria | SC | 56961.61±2945.37 | 5.99±0.91 | 0.96±0.03 | 0.55±0.08 |
|  | SW | 56897.54±1794.91 | 6.43±0.56 | 0.98±0.03 | 0.59±0.05 |
|  | SN | 57635.77±1802.49 | 6.62±0.64 | 0.98±0.01 | 0.61±0.06 |
|  | SWN | 57610.88±3611.04 | 6.47±1.14 | 0.96±0.04 | 0.60±0.10 |
|  | MC | 64403.71±351.85 | 8.53±0.13 | 1.00±0.00 | 0.78±0.01 |
|  | MW | 64505.39±343.05 | 8.64±0.08 | 1.00±0.00 | 0.78±0.01 |
|  | MN | 64632.50±840.29 | 8.57±0.18 | 1.00±0.00 | 0.78±0.02 |
|  | MWN | 64308.96±281.16 | 8.54±0.09 | 1.00±0.00 | 0.78±0.01 |
| Eukaryon | SC | 2221.79±309.14 | 4.89±0.60 | 0.97±0.02 | 0.67±0.07 |
|  | SW | 2083.47±671.63 | 4.72±1.12 | 0.94±0.08 | 0.65±0.12 |
|  | SN | 2107.03±397.26 | 4.71±0.87 | 0.95±0.03 | 0.65±0.09 |
|  | SWN | 2282.47±134.03 | 4.76±0.43 | 0.96±0.01 | 0.65±0.05 |
|  | MC | 2319.52±81.96 | 4.46±0.56 | 0.91±0.05 | 0.60±0.07 |
|  | MW | 2245.70±126.17 | 4.21±0.56 | 0.90±0.07 | 0.58±0.07 |
|  | MN | 2278.52±147.87 | 4.32±0.69 | 0.91±0.05 | 0.59±0.08 |
|  | MWN | 2182.45±155.50 | 4.33±0.31 | 0.94±0.02 | 0.60±0.04 |


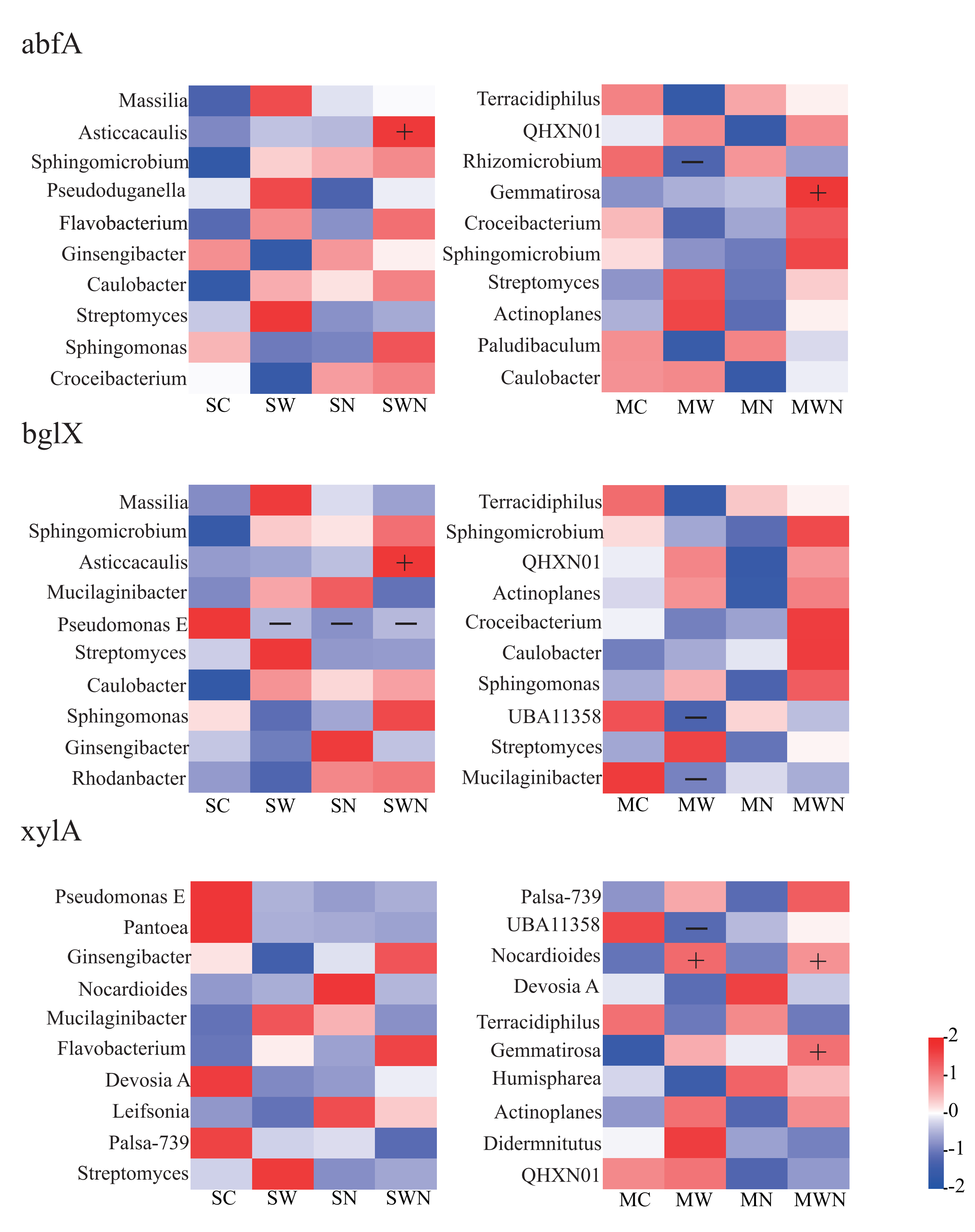


**Figure S1. Heatmap of contribution of carbon degradation gene species*.***


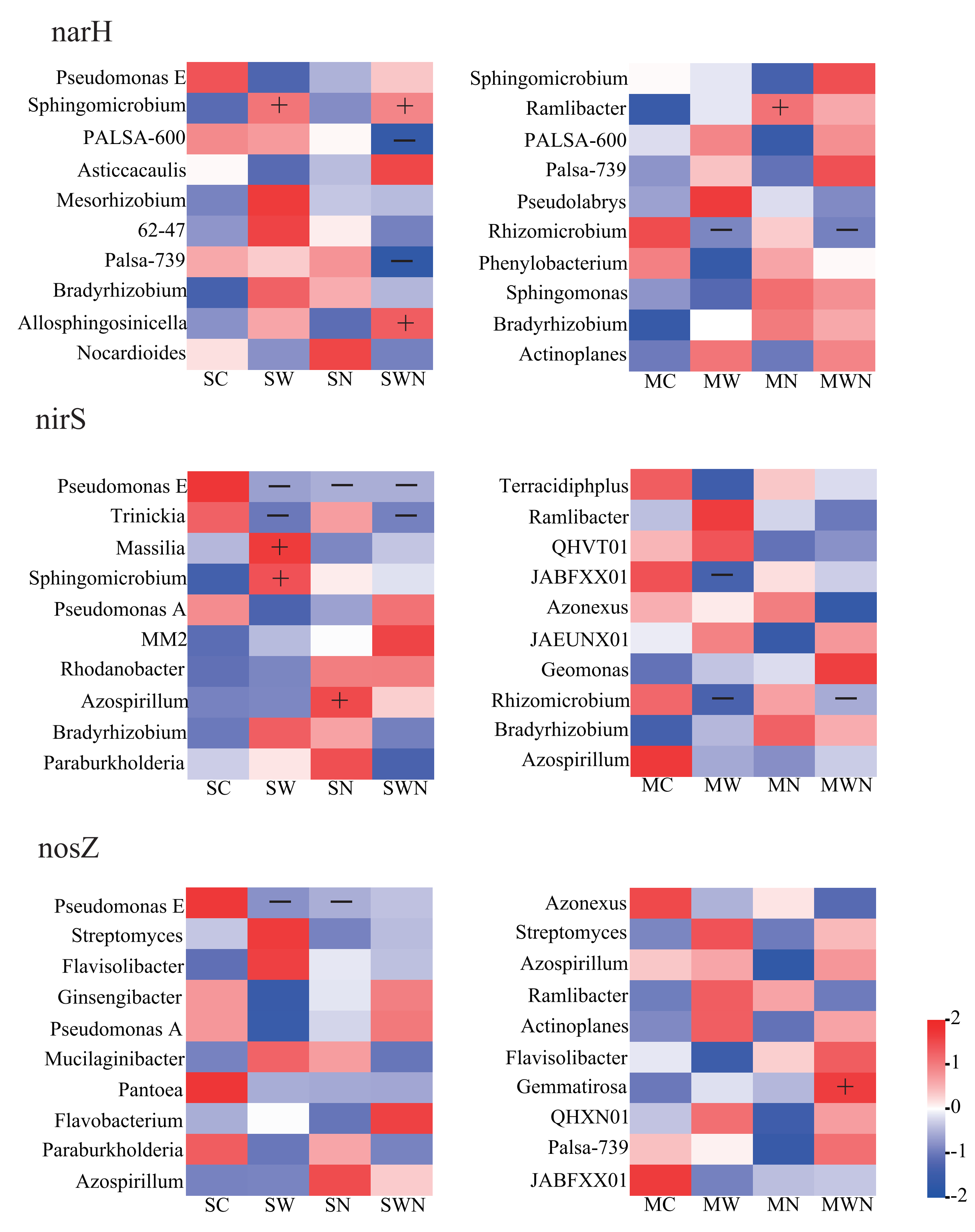


**Figure S2. Heatmap of contribution of denitrification gene species*.***


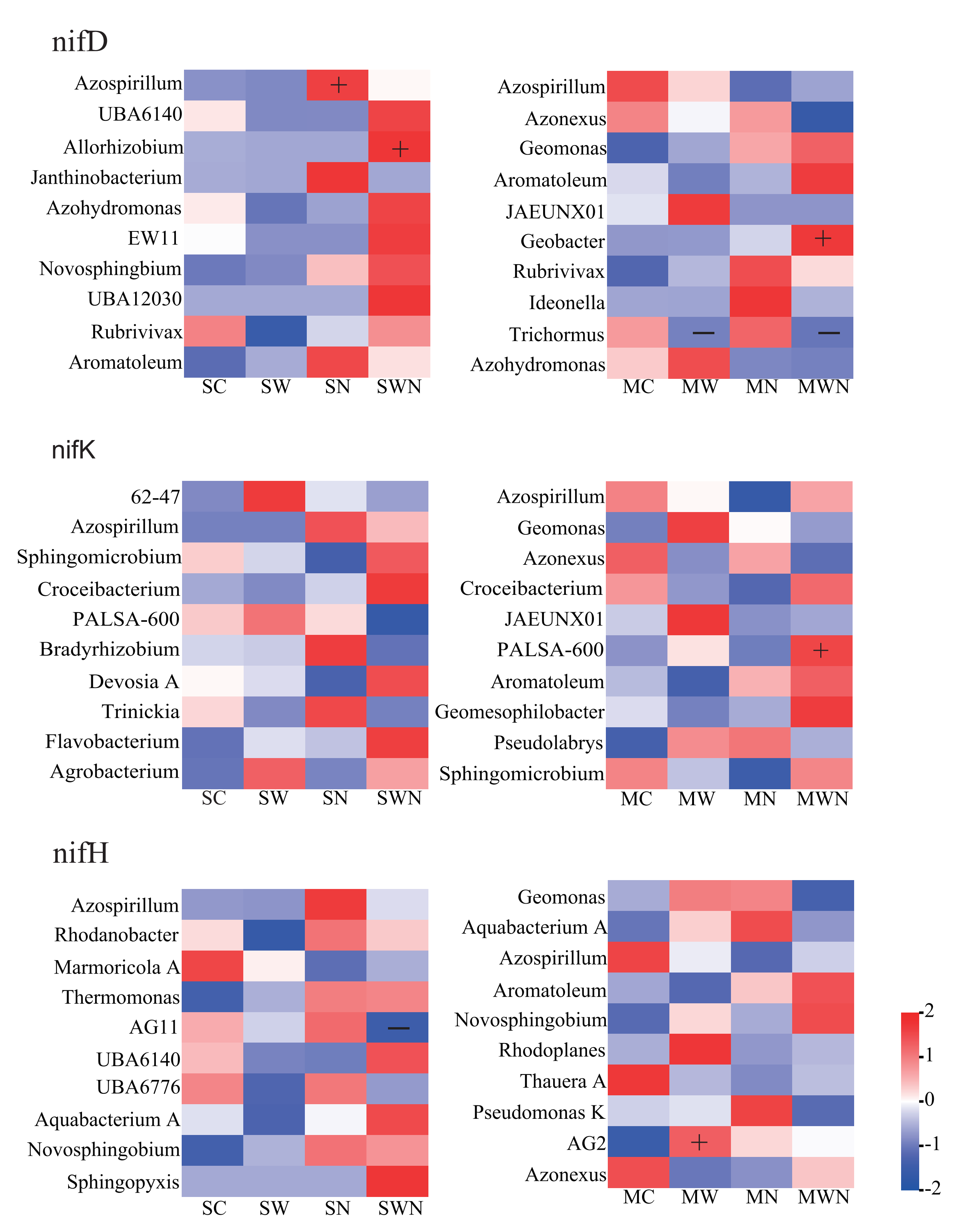


**Figure S3. Heatmap of contribution of nitrogen fixation gene species*.***


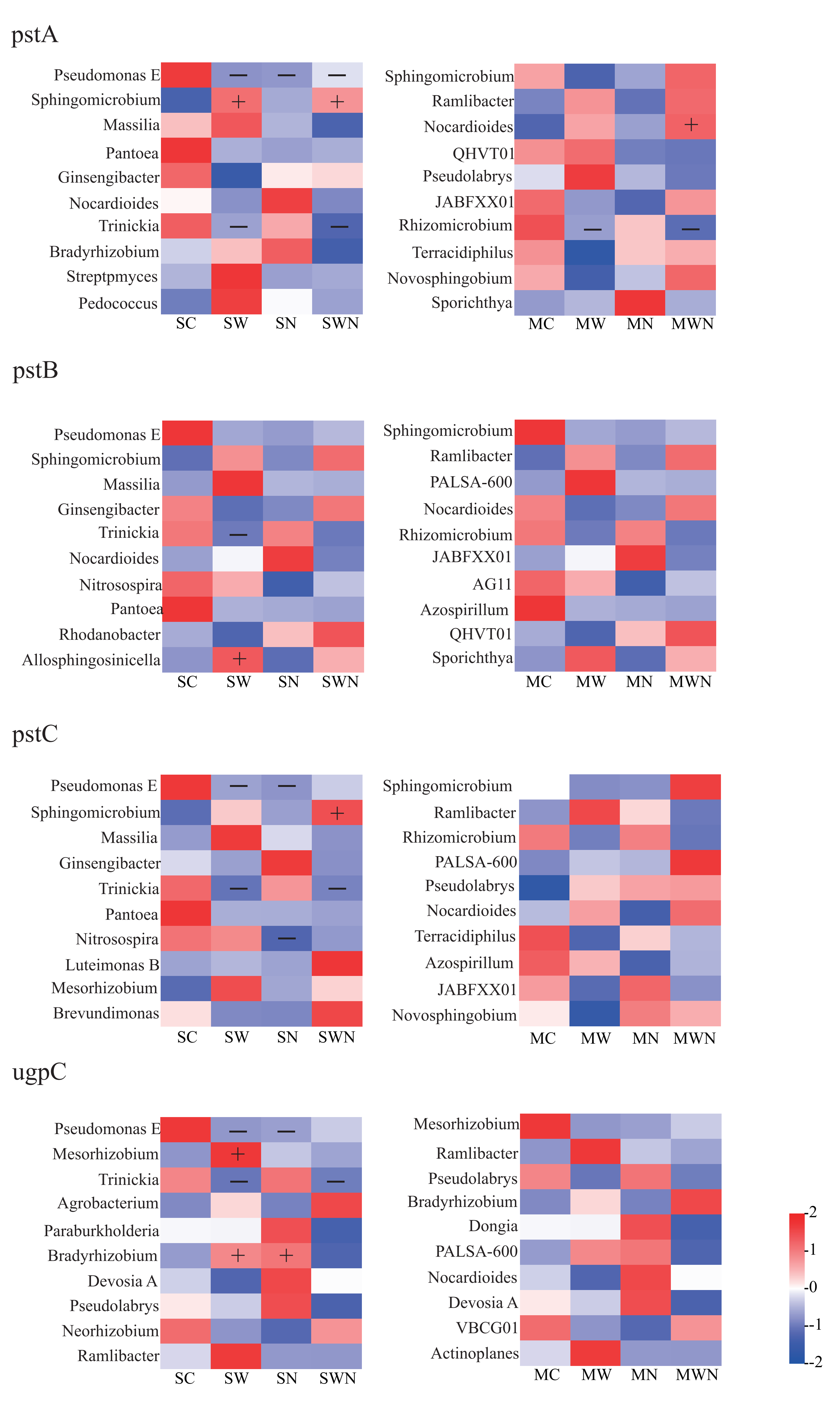


**Figure S4. Heatmap of contribution of phosphorus transport gene species*.***


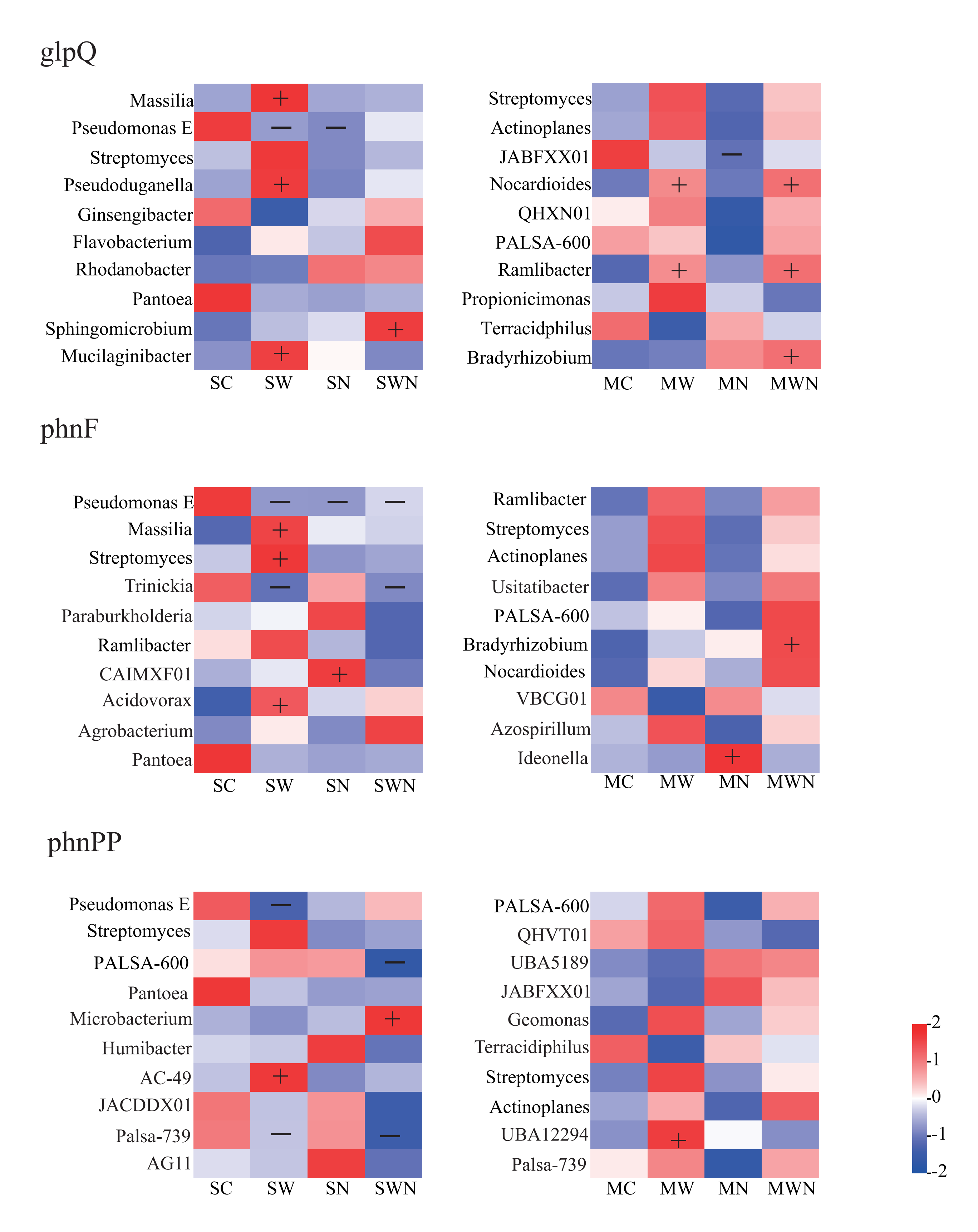


**Figure S5. Heatmap of contribution of organic phosphorus mineralization gene species*.***
